# High Concentration of an ISS-N1-Targeting Antisense Oligonucleotide Causes Massive Perturbation of the Transcriptome

**DOI:** 10.3390/ijms22168378

**Published:** 2021-08-04

**Authors:** Eric William Ottesen, Diou Luo, Natalia Nikolaevna Singh, Ravindra Narayan Singh

**Affiliations:** Department of Biomedical Sciences, Iowa State University, Ames, IA 50011, USA; eottesen@iastate.edu (E.W.O.); diouluo@iastate.edu (D.L.); natalias@iastate.edu (N.N.S.)

**Keywords:** spinal muscular atrophy, SMA, survival motor neuron, SMN, ISS-N1, antisense oligonucleotide, off-target effect, splicing, nusinersen, Spinraza

## Abstract

Intronic splicing silencer N1 (ISS-N1) located within *Survival Motor Neuron 2* (*SMN2*) intron 7 is the target of a therapeutic antisense oligonucleotide (ASO), nusinersen (Spinraza), which is currently being used for the treatment of spinal muscular atrophy (SMA), a leading genetic disease associated with infant mortality. The discovery of ISS-N1 as a promising therapeutic target was enabled in part by Anti-N1, a 20-mer ASO that restored *SMN2* exon 7 inclusion by annealing to ISS-N1. Here, we analyzed the transcriptome of SMA patient cells treated with 100 nM of Anti-N1 for 30 h. Such concentrations are routinely used to demonstrate the efficacy of an ASO. While 100 nM of Anti-N1 substantially stimulated *SMN2* exon 7 inclusion, it also caused massive perturbations in the transcriptome and triggered widespread aberrant splicing, affecting expression of essential genes associated with multiple cellular processes such as transcription, splicing, translation, cell signaling, cell cycle, macromolecular trafficking, cytoskeletal dynamics, and innate immunity. We validated our findings with quantitative and semiquantitative PCR of 39 candidate genes associated with diverse pathways. We also showed a substantial reduction in off-target effects with shorter ISS-N1-targeting ASOs. Our findings are significant for implementing better ASO design and dosing regimens of ASO-based drugs.

## 1. Introduction

Antisense oligonucleotide (ASO)-based modulation of transcript levels has emerged as a formidable tool for the treatment of a growing number of human diseases [1,2,3]. Potentially any transcript within a cell can be targeted by an ASO. The desired outcomes of transcript targeting by an ASO include manipulation of pre-mRNA splicing, degradation of specific transcripts, alteration of transcription, and suppression of translation [1]. Therapeutic ASOs can incorporate a variety of chemical modifications to enhance their efficacies in vivo [1], with limitless potential for further ASO improvement. In the case of an ASO-directed manipulation of pre-mRNA splicing, selection of a target sequence plays an important role. For example, when the goal is to promote the inclusion of a coding exon, such as the case with *SMN2* exon 7 linked to spinal muscular atrophy (SMA), an ideal target must be a negative regulatory element, which is accessible and located within an intron [4]. However, identification of intronic targets that exert a strong stimulatory or inhibitory effect on pre-mRNA splicing continues to be an arduous task. Nusinersen, the first FDA-approved drug for SMA, remains the sole success story of an ASO-based approach, where the level of a fully functional protein is increased by substantially restoring inclusion of an exon that otherwise would be skipped [3,4,5,6]. While an ASO-based approach to manipulate splicing has vast therapeutic potential, the specificity of the approach is not guaranteed due to off-target effects caused by ASO “tolerance” for mismatched base pairing during annealing and by the ASO chemistry itself [7]. Understanding the nature of off-target effects caused by a therapeutic ASO, particularly when high concentrations are used, has significance for improving dosing regimens and treatment strategies. 

SMA is a broad-spectrum disease and a leading genetic cause of infant mortality [8]. In more than 95% of instances, SMA is caused by low levels of the Survival Motor Neuron (SMN) protein due to deletions of or mutations in the *SMN1* gene [9]. SMN is a multifunctional protein involved in most aspects of cellular metabolism, such as DNA damage repair, transcription, snRNP biogenesis, splicing, translation, selenoprotein synthesis, stress granule formation, macromolecular trafficking, signaling pathways, and cytoskeletal dynamics [10,11,12,13,14,15,16]. Therefore, low levels of SMN affect most tissues, including bone, brain, gastrointestinal tract, heart, kidney, liver, lung, muscle, ovary, spleen, spinal cord, and testis [8]. SMN directly interacts with transcripts, and RNA-SMN interactions have been implicated in tissue-specific regulation of translation [17,18,19,20]. *SMN2*, a nearly identical copy of *SMN1*, cannot compensate for the loss of *SMN1* due to predominant skipping of exon 7 [21,22]. Of note, both *SMN* genes harbor exceptionally high numbers of Alu elements and generate a diverse repertoire of transcripts, including multiple alternatively spliced mRNAs, circular RNAs, and long non-coding antisense RNAs [23,24,25,26,27,28,29,30,31,32]. However, functions of most *SMN* transcript variants remain unknown. The absence of exon 7 in *SMN2* mRNA leads to the production of SMNΔ7, a truncated unstable protein [33]. Considering *SMN2* is universally present in SMA patients, the correction of *SMN2* exon 7 splicing has emerged as one of the most promising avenues for the treatment of the disease [4]. Nusinersen (Spinraza), Zolgensma (gene therapy), and risdiplam are three currently approved SMA drugs [6,34,35]. Two of them, nusinersen (an ASO) and risdiplam (a small molecule), are based on the restoration of *SMN2* exon 7 inclusion [6,35]. An intronic splicing silencer, ISS-N1, located within intron 7 serves as the target for nusinersen [6]. The mechanism by which an ISS-N1-targeting ASO promotes inclusion of *SMN2* exon 7 appears to be complex, as it involves structural rearrangements, potential displacement of negative factors, and recruitment of positive factors [36]. Discovered in 2004 and first reported in 2006, ISS-N1 remains the most studied antisense target for splicing correction in the context of a human disease [5,6,37]. 

**Figure 1:**
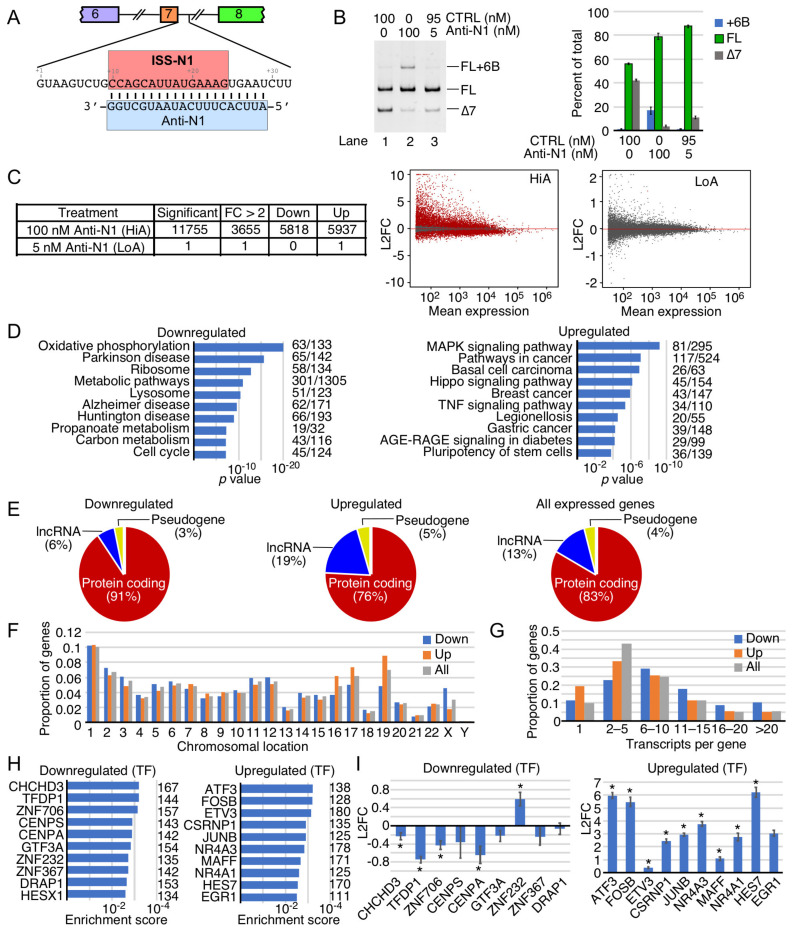
Gene expression after treatment with Anti-N1 ASO. (**A**). Schematic of Anti-N1 and its intronic target sequence ISS-N1. Exons are depicted as colored-numbered boxes, and introns are depicted as broken lines. The sequence immediately downstream of exon 7 is depicted below. Nucleotide numbering starts from the first position of intron 7. ISS-N1 is boxed in red and Anti-N1 ASO is marked with a blue box. Black lines show base pairing between Anti-N1 and its target sequence. (**B**). Alternative splicing of *SMN2* exon 7 after treatment with the indicated concentrations of Anti-N1. Left panel: representative ethidium bromide-stained gel depicting semiquantitative RT-PCR of *SMN2* (from exon 6 to exon 8). ASO concentrations are provided at the top of the gel. Splice isoform identity is provided at the right side of the gel. Right panel: densitometric quantification of RT-PCR results. Error bars represent the standard error of the mean. *n* = 3. Abbreviations: CTRL, control ASO; FL, canonical full-length isoform. (**C**). Overview of differential expression analysis of RNA-Seq performed on Anti-N1-treated GM03813 SMA patient fibroblasts. Left panel: Overall summary table describing the number of genes with altered expression after Anti-N1 treatment as compared to the nontargeting control ASO. “Significant” indicates genes with Benjamini and Hochberg adjusted *p* values (adj. *p*) < 0.05. FC > 2 indicates genes with more than 2-fold up- or downregulation. Right two panels: MA plot depicting gene expression changes upon cell treatment with Anti-N1. Y axis represents the log_2_ fold change in transcript levels in Anti-N1 treated cells as compared with the nontargeting control ASO. X axis represents the mean expression in normalized read counts per gene. Red dots indicate genes with significantly altered expression levels (adj. *p* < 0.05). Grey dots indicate unchanged genes. Abbreviations: L2FC, log_2_ fold change; HiA, 100 nM anti-N1 treatment; LoA, 5 nM anti-N1 treatment. (**D**). The top 10 enriched Kyoto Encyclopedia of Genes and Genomes (KEGG) pathways among downregulated (left panel) and upregulated (right panel) genes. Pathway names are provided at the left side, and number of affected genes/total genes in each pathway are indicated at the right side. X axis represents *p* value of enrichment. (**E**). Proportion of genes coding for mRNAs, lncRNAs, and pseudogene transcripts among downregulated (left), upregulated (middle), and all expressed genes in GM03813 cells (right). (**F**). The chromosomal distribution of significantly downregulated (blue), upregulated (orange), and all expressed genes (gray). (**G**). The proportion of significantly downregulated (blue), upregulated (orange) and all expressed genes (gray) that encode different numbers of alternative transcripts. (**H**). The 10 most significant transcription factors whose targets are enriched in genes downregulated (left panel) and upregulated (right panel) by HiA treatment. Each transcription factor is indicated at the left side, and number of significantly affected target genes is indicated at the right side. X axis indicates statistical significance. (**I**). The relative expression levels of transcription factors described in H after HiA treatment. Y axis represents L2FC compared to control. *—adj. *p* < 0.05.

ISS-N1 is a 15-nucleotide-long sequence spanning from the 10th to 24th positions of *SMN2* intron 7 (Figure 1A) [37]. Sequestration of the first nucleotide of ISS-N1 (10th intronic position) has been found to be essential for the stimulatory effect of an ASO on *SMN2* exon 7 splicing [38,39,40]. The size of the ISS-N1-targeting ASOs used in most studies varies between 15 and 25 nucleotides [41,42,43]. Longer ASOs that anneal to ISS-N1 and the immediate downstream sequences show a robust stimulatory effect on *SMN2* exon 7 inclusion [44,45]. Anti-N1, first reported in 2006, is a 20-mer ASO incorporating a phosphorothioate backbone along with 2′-O-methyl ribose sugar modifications (abbreviated as “2′OMe” ASO) (Figure 1A) [37,42]. Anti-N1 can stimulate *SMN2* exon 7 splicing at concentrations as low as 1 nM, the lowest reported effective concentration for any ISS-N1 targeting ASO [42]. However, ~100 nM of this ASO was required to have a substantial effect on *SMN2* exon 7 inclusion [42]. Even higher concentrations have been used to achieve the full restoration of *SMN2* exon 7 inclusion by other ISS-N1-targeting ASOs that carried phosphorothioate-modified backbones in conjunction with 2′-O-methoxyethyl-modified sugar (abbreviated as “MOE” ASO) as well as morpholino ASOs [43,44,45]. In vivo studies in SMA mouse models generally employ very high ASO doses (25 to 160 mg/kg of body weight) that fall in the high micromolar concentration range [43,44,45,46,47]. The nature and the extent of off-target effects caused by high concentrations of any of the ISS-N1-targeting ASOs have not been examined yet. For the sake of convenience, we used several acronyms that are described in Table 1.

## 2. Results

### 2.1. Transcriptome-Wide Effect of Anti-N1

To determine the transcriptome-wide effect of an ISS-N1-targeting ASO, we performed RNA-Seq on transcripts of SMA type I patient-derived fibroblasts (GM03813 cells) treated with either 5 nM Anti-N1 (LoA) or 100 nM Anti-N1 (HiA) for 30 h. We used 10mer ASO as control that had no effect on *SMN2* exon 7 splicing compared to the untransfected GM03813 cells. Of note, GM03813 cells carry only the *SMN2* gene. These cells have been widely used for the screening of splicing-modulating compounds. As expected, both LoA and HiA produced the intended increase in *SMN2* exon 7 inclusion, although the effect was more pronounced at HiA (Figure 1B). HiA also promoted inclusion of *SMN2* exon 6B (Figure 1B). Of note, inclusion of *SMN2* exon 6B changes the critical C-terminus of SMN; the resulting SMN6B protein has a slightly higher stability than SMNΔ7 [26]. However, considering exon 6B-containing transcripts are substrates of nonsense-mediated decay (NMD), the steady-state levels we observed are probably lower than their actual expressions [26]. RNA-Seq of HiA-treated samples revealed significant alterations in the expression of 11,755 genes, with transcription levels of 3655 genes being changed by more than 2-fold (Figure 1C, Supplementary Tables S1 and S2). According to MA plot based on log_2_ fold change (L2FC), HiA triggered a disproportionate upregulation of genes that are expressed at low levels. However, in case of genes with mean expression of 10^3^ normalized counts and higher, the effect of HiA was almost evenly split between upregulated and downregulated ones (Figure 1C). In contrast, LoA hardly produced any effect on the transcriptome. In fact, only one gene, the lncRNA AL021155.5, was upregulated in LoA-treated samples (Figure 1C). 

While discussion of all cellular functions and pathways impacted by HiA is beyond the scope of this study, we focused on important functions and representative genes affected by HiA. Critical processes associated with some of the most strongly upregulated genes (Supplementary Table S1) include transcription (*ETS2*, *KLF4*, *HEXIM1*, *IER2*, *MSC*, *MAFB*, *EGR1*, *NR4A1*, *JUNB*, *CSRNP1*, *ETV3*, *FOSB*, and *ATF3*), RNA transport (*NXF1*), cell signaling (*RHOB*, *SESN2*, *HSPA2*, and *SOCS1*), cytoskeletal dynamics (*MARCKSL1* and *ARC*), and metabolic pathways (*DYRK3*). Similarly, genes which were significantly downregulated by HiA (Supplementary Table S2) are associated with the regulation of essential functions, including transcription (*TFDP1*, *ZNF706*, and *PRMT5*), mitochondrial function (*CHCHD3*), apoptosis (*BLID*), cell signaling (*CD9*, *RAB7B*, *GBP2*, and *FANCL*), cell cycle (*DLGAP5*), cell adhesion (*CLDN11*), metabolic pathways (*METTL7A* and *FAXDC2*), synaptic activity (*SLC17A6*), extracellular matrix breakdown (*MMP1* and *MMP3*), and metal binding (*MT2A*). Interestingly, one of the highly expressed cancer-associated long non-coding RNA (lncRNA), *MALAT1*, was also significantly downregulated by HiA. *MALAT1* is generated by RNase P cleavage of the tRNA-like small non-coding RNA and lacks a canonical poly(A) tail [48]. The implicated functions of *MALAT1* are regulation of transcription and translation through sequestration of proteins and microRNAs, respectively [48]. 

To interpret the potential biological consequence of changes in the transcriptome caused by HiA treatment, we also performed overrepresentation analysis, which determines if any functional pathways were significantly enriched. The enriched downregulated genes were associated with oxidative phosphorylation, neurodegenerative disorders, ribosome biogenesis, metabolic pathways, lysosome, and cell cycle (Figure 1D). The enriched upregulated genes were associated with mitogen-activated protein kinase (MAPK) signaling pathway, cancer and Hippo signaling pathways, Advanced Glycation End Product (AGE)-Receptor for AGE (AGE-RAGE) signaling in diabetes, and pluripotency of stem cells (Figure 1D). In addition to protein-coding genes, expression of lncRNAs and pseudogenes were also impacted by HiA treatment (Figure 1E). The relative proportions of the upregulated lncRNAs and pseudogenes were higher than those of the downregulated lncRNAs and pseudogenes. Interestingly, lncRNAs constituted almost 1/5th of the total upregulated transcripts. Overall, according to our analysis, HiA treatment affected the expression of genes located on all chromosomes (Figure 1F). Noticeably, a disproportionately greater number of genes located on chromosomes 16, 17, and 19 were upregulated, while a substantially higher proportion of genes located on the X-chromosome were downregulated (Figure 1F). Our results also showed that genes expressing 5 or less RNA isoforms were upregulated (Figure 1G). Of note, RNA isoforms included transcripts generated by alternative splicing, by usage of alternative transcription start sites, or usage of alternative Poly(A) sites. For instance, genes producing a single transcript represented ~10% of all expressed genes, while they constituted ~20% of all genes upregulated by HiA treatment (Figure 1G). On the contrary, genes producing more than 20 isoforms represented ~5% of all expressed genes, but they made up ~10% of all the downregulated genes (Figure 1G). This indicates that as the complexity of transcription/splicing regulation increased, genes became more prone to downregulation by Anti-N1 treatment.

In addition, we categorized the affected genes based on their likely regulation by common transcription factors. Among the predicted top ten transcription factors associated with the downregulated genes were *CHCHD3*, *TFDP1*, *ZNF706*, *CENPS*, *CENPA*, *GTF3A*, *ZNF232*, *ZNF367*, *DRAP1*, and *HESX1* (Figure 1H). Among the predicted top ten transcription factors associated with the upregulated genes were *ATF3*, *FOSB*, *ETV3*, *CSRNP1*, *JUNB*, *NR4A3*, *MAFF*, *NR4A1*, *HES7*, and *EGR1* (Figure 1H). Barring one exception (*ZNF232*), the expression levels of the predicted transcription factors associated with the downregulated genes were also reduced (Figure 1I). However, the magnitude of this reduction did not exceed 1.6 times, suggesting the involvement of additional factors in modulating the expression of the downregulated genes. Similarly, all the predicted transcription factors associated with the upregulated genes showed increased expressions themselves (Figure 1I). Further, with the exception of *ETV3* and *MAFF*, we observed a >4-fold increase (>2 L2FC) in the levels of transcription factors associated with the upregulated genes (Figure 1I). The highest increase in expression was observed for *ATF3*, *FOSB*, and *HES7* (Figure 1I).

### 2.2. Nature of Splicing Events Impacted by Anti-N1 

**Figure 2:**
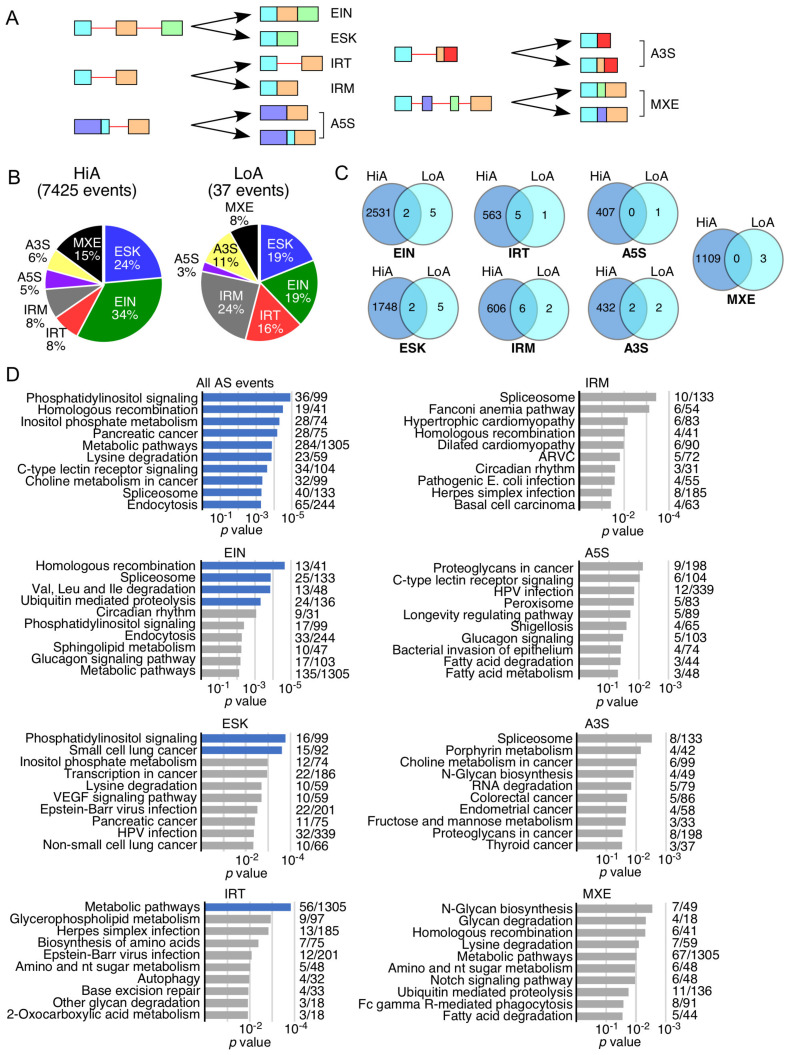
An overview of alternative splicing affected by Anti-N1 treatment. (**A**). Diagrammatic representation of each type of alternative splicing event under investigation. Exons are represented by colored boxes, while introns by red lines. Abbreviations: EIN, exon inclusion; ESK, exon skipping; IRT, increased intron retention; IRM, improved intron removal; A5S, alternative 5′ splice site; A3S, alternative 3′ splice site; MXE, mutually exclusive/mixed exons. (**B**). Pie charts depicting the types and relative proportion of alternative splicing events affected by Anti-N1 treatment. The treatment type and total number of significant splicing events affected by it is indicated at the top. (**C**). Venn diagrams comparing similarities and differences between the events triggered by HiA and LoA. (**D**). The top 10 enriched KEGG pathways among genes containing all splicing events and each type of alternative splicing event after HiA treatment. Pathway names are provided at the left side, number of affected genes/total genes in each pathway are indicated at the right side. Blue bars indicate pathways with highly significant (false discovery rate (FDR) <0.05) enrichment, and gray bars indicate pathways with *p* values < 0.05 but FDR > 0.05. X axis represents *p* value of enrichment.

We analyzed the effect of Anti-N1 on pre-mRNA splicing. In particular, we examined seven types of splicing events changed by Anti-N1 treatment: enhanced exon inclusion (EIN), enhanced exon skipping (ESK), increased intron retention (IRT), improved intron removal (IRM), alternative 5′ splice site (5′ss) selection (A5S), alternative 3′ss selection (A3S), and aberrant mixed splicing (MXE) that included splicing of mutually exclusive exons (Figure 2A). While 7425 splicing events were altered by HiA, only 37 splicing events were affected by LoA (Figure 2B, Supplementary Tables S3–S16). The order of HiA-affected splicing events was: EIN > ESK > MXE > IRT = IRM > A3S > A5S (Figure 2B). EIN and ESK together accounted for a whopping 58% of all splicing events altered by HiA treatment. MXE, IRT, IRM, A3S, and A5S represented 15%, 8%, 8%, 6%, and 5% of the total splicing events impacted by HiA, respectively (Figure 2B). In the case of LoA treatment, the order of altered splicing events was: IRM > EIN = ESK > IRT > A3S > MXE > A5S (Figure 2B). In contrast to a relatively low proportion of IRM caused by HiA, IRM was the most frequent event caused by LoA, representing ~24% of the total aberrant splicing events (Figure 2B). Six out of eight IRM, five out of six IRT, and two out of four A3S events affected by LoA overlapped with those affected by HiA (Figure 2C). However, a significantly smaller overlap between LoA and HiA treatments was observed for EIN and ESK events (Figure 2C). None of the MXE and A5S changes were the same between LoA and HiA treatments (Figure 2C). 

We performed overrepresentation analysis to determine whether any functional pathways were significantly enriched among genes whose pre-mRNA splicing was affected by Anti-N1 treatments. When all splicing events were analyzed together, at least ten pathways/processes were found to be impacted by the treatments with high confidence. These included phosphatidylinositol signaling, homologous recombination, pancreatic cancer, metabolic pathways, lysine degradation, C-type lectin receptor signaling, choline metabolism in cancer, spliceosome, and endocytosis (Figure 2D). When the overrepresentation analysis was performed individually for each of the seven types of affected splicing events, multiple pathways/processes were found to be impacted as well, but with low confidence. However, our overrepresentation analysis did capture a few pathways/processes impacted with high confidence in three types of splicing, namely EIN, ESK, and IRT (Figure 2D). 

Consistent with the largest number of the aberrant splicing events, EIN showed the greatest number of pathways/processes impacted by Anti-N1 treatments with high confidence. These pathways/processes included homologous recombination, spliceosome, Val/Leu/Ile degradation, and ubiquitin-mediated proteolysis (Figure 2D). ESK had two significantly impacted pathways/processes: phosphatidylinositol signaling and small cell lung cancer (Figure 2D). In case of IRT, metabolic pathway was the only one affected with high confidence (Figure 2D). For the remaining types of alternative splicing events, no pathway crossed the threshold of high significance. 

### 2.3. Analysis of Exons Undergoing Enhanced Inclusion Caused by Anti-N1

**Figure 3:**
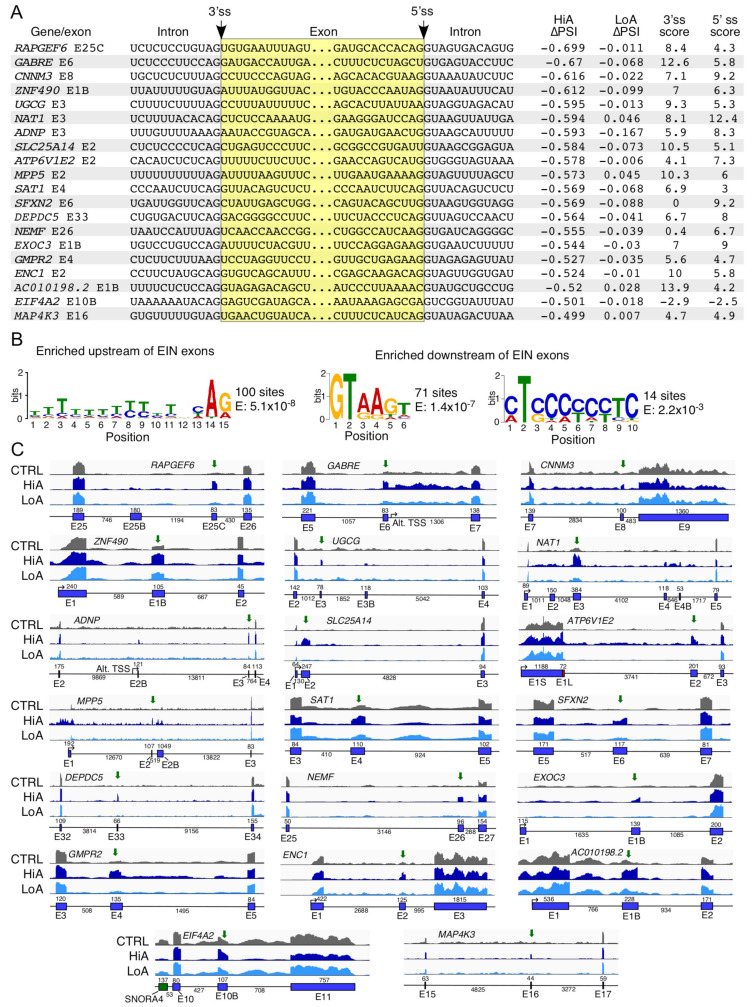
Increased exon inclusion caused by Anti-N1 treatment. (**A**). The sequence context of 20 candidate exons exhibiting greatly increased inclusion upon Anti-N1 treatment; 12 nt-long sequences on either side of the 3′ss and 5′ss of each affected exon are provided. Exonic sequences are highlighted by a yellow box. The 3′ss and 5′ss are indicated by arrows. At the right of each sequence, the change in skipping (∆PSI) under HiA and LoA treatment are provided, along with the predicted strength of each 3′ss and 5′ss. (**B**). Enriched sequence motifs identified by MEME. Left panel: the most enriched motif within 50 nucleotides upstream of the 3′ss of the affected exons, corresponding to the polypyrimidine tract and the 3′ss sequence. Middle and right panel: two motifs enriched downstream of the 5′ss of the affected exons. The first motif matches the consensus 5′ss and the second represents a C- and U-rich motif of unknown significance. *n* = 100 sequences. (**C**). Genomic views of several candidate exons whose inclusion was increased upon Anti-N1 treatment. The ASO treatments are indicated at the left side. The name of the host gene is provided at the top of each panel; exon locations and their names are shown below each panel. Exons are depicted by blue boxes, introns by black lines. Exon sizes are indicated above each exon, whereas, intron sizes are indicated below each intron. For each genomic region an exon whose inclusion is increased by Anti-N1 treatments is indicated with a green arrow. Transcription start sites are indicated with arrows pointing in the direction of transcription. A snoRNA derived from an intronic sequence in *EIF4A2* is indicated by a green box and marked. Abbreviations: CTRL, the nontargeting control ASO.

Increase in exon inclusion was the most frequent aberrant splicing event triggered by HiA with 2536 events (Supplementary Table S3), and one of the most frequent events triggered by LoA with seven events (Supplementary Table S4). We examined 20 representative exons that underwent enhanced inclusion (EIN) under HiA treatment (Figure 3A). Importantly, many genes associated with the aberrant EIN events described here are linked to pathological conditions, including cancer (*NAT1*, *NEMF*, *GMPR2*, *ENC1*, *MAP4K3*, and *EIF4A2*), epilepsy (*GABRE* and *DEPDC5*), metabolic disorder (*UGCG*), schizophrenia/personality disorder (*RAPGEF6*), mental disorder (*ATP6V1E2*), retinitis pigmentosa (*MPP5*), keratosis (*SAT1*), hypertension (*SFXN2*), autism (*ADNP*), adult-onset Still’s disease (MAP4K3), and pulmonary disorder (*CNNM3*). Several genes in the list are associated with important cellular functions, including translation (*NEMF*), organelle biogenesis (*EXOC3*), cytoskeletal dynamics (*ENC1*), membrane transport (*SFXN2*), immune response (MAP4K3), energy metabolism (*SLC25A14* and *ATP6V1E2*), cell signaling (*MAP4K3* and *DEPDC5*), and nucleic acid binding (*EIF4A2* and *ZNF490*). In addition, one of the genes affected by aberrant EIN, AC010198.2, codes for a lncRNA with unknown function. Exons whose inclusion was increased by Anti-N1 displayed varied contexts in terms of their sizes, sequence compositions, and their flanking intronic sequences. We analyzed representative splice sites involved in EIN events and scored them according to their adherence to the consensus 5′ss and 3′ss (Figure 3A). We observed an average score of 6.8 for the 3′ss and 6.2 for the 5′ss of exons listed in Figure 3A. In comparison, average scores for the constitutive 5′ss and 3′ss happened to be 8.1 and 7.9, respectively. Only one exon, *NAT1* exon 3, had an average or higher score for both splice sites (Figure 3A). Such suboptimal scores suggest that the exons might be poorly recognized/included during pre-mRNA splicing. We also analyzed 100 sequences to look for motif enrichment within the polypyrimidine tract, the 3′ss and the 5′ss. The emerged motifs were largely in conformity with the consensus 3′ss and 5′ss motifs associated with U2 type introns (Figure 3B). Interestingly, we found an enrichment of a CU-rich motif downstream of the 5′ss of the effected exons (Figure 3B). Given that there is little complementarity between this sequence and Anti-N1, we predict that this motif represents a cis-element targeted by an Anti-N1 regulated factor or part of a more complex structure that is disrupted by Anti-N1 binding nearby.

Our analysis also captured some instances of concomitant splicing changes associated with the EIN events. For instance, increase in inclusion of *GABRE* exon 6, *GMPR2* exon 4, *ATP6V1E2* exon 2, and *ZNF490* exon E1B was accompanied by retention of the downstream and/or upstream intron (Figure 3C). We hypothesize that this intron retention could be due to Anti-N1 blocking of positive elements or the splice sites of the upstream and/or downstream exon. In other cases, namely *SAT1* exon 4, *UGCG* exon 3, and *EIF4A2* exon 10B, the EIN event was accompanied by an increase in removal of the downstream and/or upstream intron (Figure 3C). This is likely to happen when a negative splicing regulatory element is blocked by Anti-N1. In the case of *GABRE*, increase in inclusion of exon 6 was accompanied by a significant increase of intronic reads; however, several simultaneous events, such as the usage of an alternative transcription start site downstream of exon 6 as well as the usage of several unannotated cryptic splice sites, complicated result interpretation (Figure 3C). In specific instances, when inclusion of the second exon was increased by HiA, as is the case with *MPP5* exon 2, the size of the first exon appeared to become bigger due to an increase of reads mapping upstream of the annotated transcription start site (Figure 3C). We hypothesize that Anti-N1 might interact with the promoter region of the gene in question, triggering a change in the transcription start site selection. 

### 2.4. Analysis of Exons Undergoing Enhanced Skipping Caused by Anti-N1

**Figure 4:**
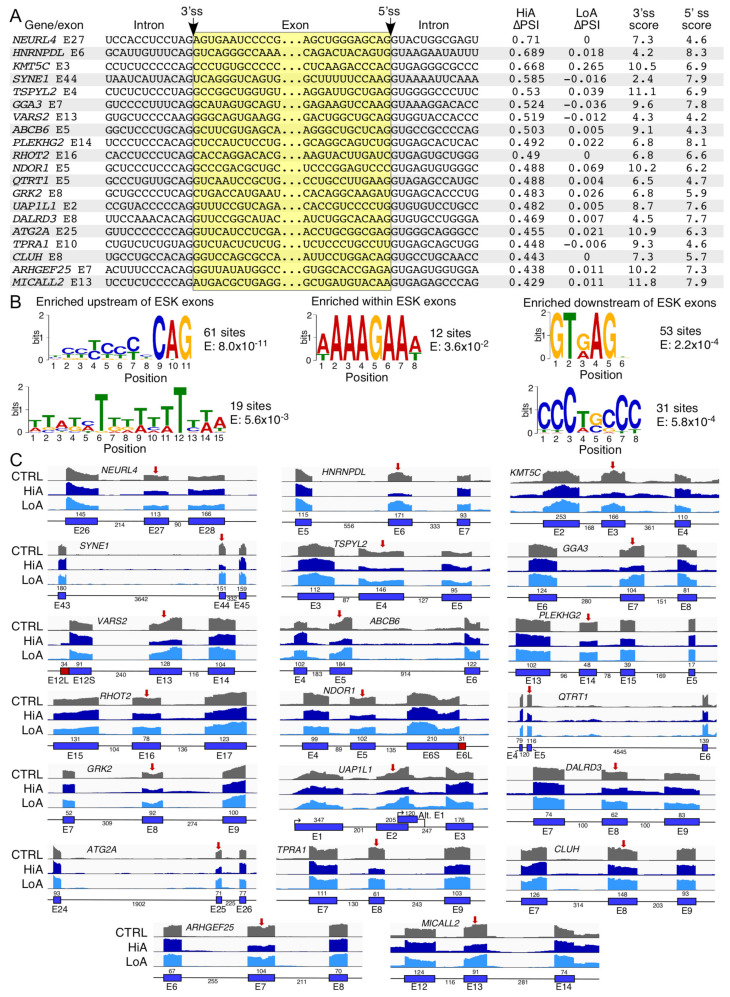
Increased exon skipping caused by Anti-N1 treatment. (**A**). The sequence context of 20 candidate exons exhibiting greatly increased skipping upon Anti-N1 treatment. Table contents and annotation are the same as in Figure 3A. (**B**). Enriched sequence motifs identified by MEME. Left panels: the two most enriched motifs within 50 nucleotides upstream of the 3′ss of affected exons, both corresponding to the polypyrimidine tract and 3′ss sequence. Middle panel: the most enriched motif within the affected exons. Right panels: two motifs enriched downstream of the affected exons. The top motif corresponds to the consensus 5′ss, and the bottom one corresponds to a C-rich motif. *n* = 100 sequences. (**C**). Genomic views of several candidate exons whose skipping is increased upon Anti-N1 treatment. For each genomic region an exon whose skipping is increased by Anti-N1 treatment is specified with a red arrow. Alternative 5′ss and 3′ss are indicated with red extensions to the original exon. The rest of the labeling and coloring is the same as in Figure 3C.

Induction of exon skipping (ESK) was the second most frequently occurring aberrant splicing event triggered by HiA. There were 1750 ESK events induced by HiA treatment (Supplementary Table S5) and 7 events induced by LoA treatment (Supplementary Table S6). Similar to EIN events, we examined 20 representative ESK events caused by HiA treatment (Figure 4A). Genes affected by aberrant ESK are linked to the diverse pathological conditions that include Angelman syndrome (*NEURL4*), muscular dystrophy (*HNRNPDL*), hyperoxaluria (*KMT5C*), spinocerebellar ataxia (*SYNE1* and *ATG2A*), cancer (*TSPYL2*), nephrotic syndrome (*GGA3*), combined oxidative phosphorylation deficiency (*VARS2*), dyschromatosis universalis hereditaria 3 (*ABCB6*), dystonia (*PLEKHG2*), 3-Methylglutaconic Aciduria (*RHOT2*), hypoxia (*NDOR1*), autism (*QTRT1*) Alzheimer’s disease (*GRK2*), metabolic disorders (*UAP1L1*), epilepsy (*DALRD3*), hyperhidrosis (*TPRA1*), Hirschsprung disease (*CLUH*), atrophic rhinitis (*ARHGEF25*) and retinitis pigmentosa (*MICALL2*). These genes are also involved in several important cellular processes, such as transcription/chromatin remodeling (*HNRNPDL*, *KMT5C* and *TSPYL2*), alternative splicing (*HNRNPDL*), translation (*CLUH*), centriolar homeostasis (*NEURL4*), nucleocytoplasmic interactions (*SYNE1*), cellular trafficking (*GGA3* and *MICALL2*), tRNA biogenesis (*VARS2*, *QTRT1* and *DALRD3*), mitochondrial function (*ABCB6* and *RHOT2*), signaling pathways (*PLEKHG2*, *RHOT2*, *GRK2*, *TPRA1* and *ARHGEF25*), energy metabolism (*NDOR1*) and autophagy (*ATG2A*). 

Similar to the EIN events, the context of exons undergoing aberrant ESK varied with regards to their sizes, the exon sequences, and their flanking intronic sequences. The 5′ss of almost all affected exons had suboptimal scores (Figure 4A). In most cases, the score of their 3′ss was suboptimal as well. We analyzed the 20 candidate exons and their flanking intronic sequences for complementarity to Anti-N1. Three exons in particular had extensive complementarity to Anti-N1; they are *SYNE1* exon 44, *VARS2* exon 13, and *TSPYL2* exon 4. Anti-N1 can potentially bind an internal site in *SYNE1* exon 44, blocking a positive cis-element or competing with a splicing factor for binding (Supplementary Figure S1A). Interestingly, Anti-N1 may bind to the sequence immediately downstream of the 3′ss of *VARS2* exon 13 and can block the 5′ss of *TSPYL2* exon 4. We performed an analysis of 100 sequences that revealed the presence of two intronic motifs upstream, a purine-rich motif within and two intronic motifs downstream of the effected exons respectively (Figure 4B). Consistent with the suboptimal splice sites, a sizeable fraction of sequences deviated from the consensus 3′ss and 5′ss motifs associated with the U2 type introns (Figure 4B). The enriched intronic C- and U-rich motifs upstream of the skipped exons could have complementarity with the G- and A-rich 3′ end of Anti-N1, while the purine-rich motif within these exons had partial complementarity with the U-rich 5′ end of Anti-N1 (Supplementary Figure S1B), suggesting a mechanistic basis of how this ASO might trigger exon skipping. 

In the majority of instances, the ESK events were accompanied by an efficient removal of the flanking introns, suggesting that Anti-N1 promoted pairing of the 5′ss of the upstream exon with the 3′ss of the downstream exon. Of note, with enhanced exon skipping, we observed only limited accompanying splicing events. For instance, an increase in skipping of *DALRD3* exon 8 and *KMT5C* exon 3 was accompanied by retention of the upstream and/or downstream intron (Figure 4C). It is likely that these associated events are not mechanistically related and might represent a mix of splicing outcomes caused by Anti-N1 targeting multiple sites. 

### 2.5. Analysis of Intron Retention Events Induced by Anti-N1 Treatment

**Figure 5:**
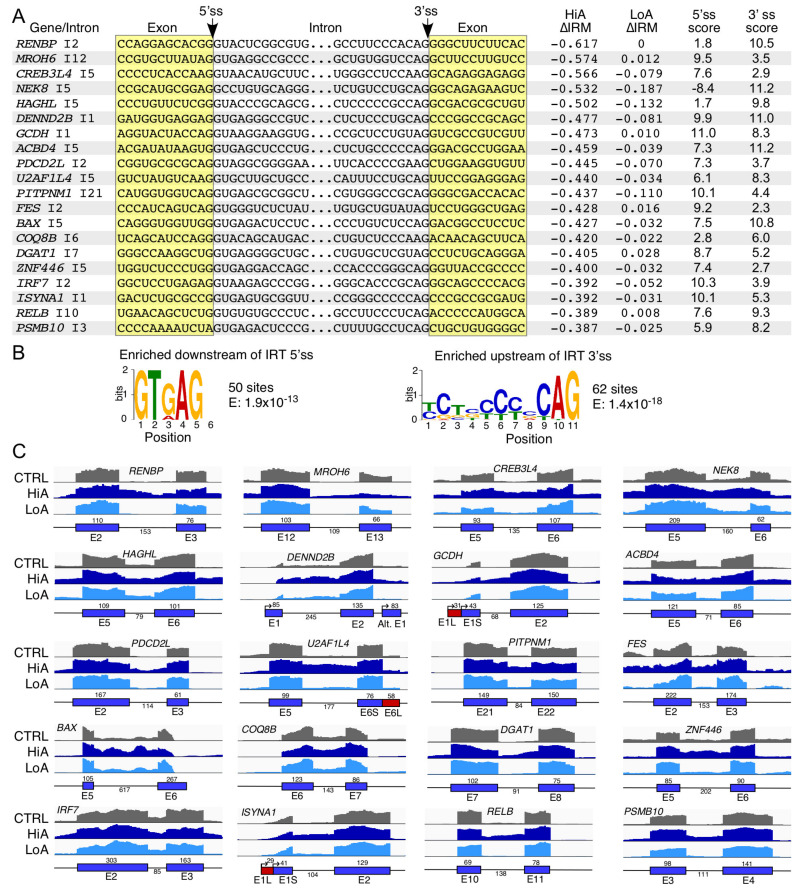
Increased intron retention caused by Anti-N1 treatment. (**A**). The sequence context of 20 candidate introns exhibiting greatly increased retention upon Anti-N1 treatment; 12 nt-long sequences that surround each indicated splice site are shown. ∆IRM indicates the change in proportional intron removal vs. intron retention. Other table contents and annotation are the same as in Figure 3A. (**B**). Enriched sequence motifs identified by MEME. Left panel: the most enriched motif within 50 nucleotides downstream of the 5′ss of retained introns, which matches the first 5 nucleotides of the consensus 5′ss motif. Right panel: the most enriched motif upstream of the 3′ss of retained introns, which closely resembles the polypyrimidine tract and the 3′ss AG dinucleotide. *n* = 100 sequences. (**C**). Genomic views of several candidate introns whose retention is increased upon Anti-N1 treatment. Labeling and coloring are the same as in Figure 3C.

Intron retention (IRT) results from the inability of the 5′ss of an intron to pair with its 3′ss. Although cassette exon events were more common, IRT was a significant driver of alternative splicing in both HiA and LoA treatment (Supplementary Tables S5 and S6). We examined 20 representative IRT events caused by HiA treatment (Figure 5A). Genes associated with the IRT events described here are linked to a number of pathological conditions, including cancer (*PSMB10*, *BAX*, *FES*, *PITPNM1*, *U2AF1L4*, and *CREB3L4*), rheumatoid arthritis (*RELB*), nemaline myopathy 1 (*ISYNA1*), immunodeficiency (*IRF7*), diarrhea (*DGAT1*), nephrotic syndrome (*COQ8B*), Fundus dystrophy (*ACBD4*), glutaric acidemia I (*GCDH*), Charcot-Marie-Tooth disease (*DENND2B*), nephronophthisis (*NEK8*), non-syndromic intellectual disability (*MROH6*), and color blindness (*RENBP*). 

In addition, cellular functions associated with genes with IRT caused by HiA treatment include regulation of transcription (*ZNF446*, *RELB*, and *CREB3L4*), innate immune response (*DGAT1*), splicing (*U2AF1L4*), proteasome activity (*PSMB10*), apoptosis (*BAX* and *PDCD2L*), cell signaling (*FES*, *PITPNM1*, *PDCD2L*, *RELB*, *IRF7*, *DENND2B*, and *NEK8*), and metabolic pathways (*ISYNA1*, *DGAT1*, *COQ8B*, *ACBD4*, *GCDH*, *HAGHL*, and *RENBP*). Analysis of the 5′ and 3′ end sequences of 100 affected introns yielded motifs typical for the 5′ and 3′ss of U2 introns (Figure 5B). Absence of a U residue at the 6th intronic position in the majority of cases underscores the weak nature of these 5′ss. The enriched motif at the 3′ end of the analyzed introns was in conformity with the consensus 3′ss in ~62% incidences (Figure 5B). Consistent with the nature of the enriched motifs, either the 5′ or the 3′ss of the majority of the affected introns had suboptimal scores (Figure 5A). However, we observed no direct correlation between splice site strength and the IRT events induced by HiA. For instance, *DENND2B* intron 1 was retained despite the high scores of both the 5′ and 3′ss (Figure 5A). 

Noticeably, the introns involved in the IRT events tended to be extremely short. Of the 20 representative introns we examined, 17 were shorter than 200 nucleotides, and the longest intron was only 617 nucleotides long. In about 50% instances (see *U2AF1L4*, *PITPNM1*, *BAX*, *COQ8B*, *DGAT1*, *ISYNA1*, *ZNF446*, *RELB*, and *PSMB10*) an IRT event did not affect the removal of the adjacent introns (Figure 5C). This observation supports an intron definition model in which sequences responsible for intron removal are present within the intron itself, as the distal splice sites in the flanking exons retained their full function. Among the remaining introns, such as *NEK8* intron 5, *HAGHL* intron 5, *ACBD4* intron 5, *FES* intron 2, and *IRF7* intron 2, removal of the upstream and/or downstream introns was negatively impacted by the corresponding IRT event (Figure 5B). This is likely due to co-regulation of splicing of two adjacent introns through an exon definition model. In case of *MROH6*, we observed both the retention of intron 12 and the decrease in inclusion of the downstream exon 13 (Figure 5B). This is likely due to the targeting of two different sites by Anti-N1. While targeting of one of these sites promotes the IRT event, targeting of the other one promotes the ESK event. In case of *GCDH*, the IRT event caused by HiA was accompanied by shifting of the transcription start site to the upstream region (Figure 5B). The mechanism behind such consequences of HiA treatment is probably complex; it might involve Anti-N1 interactions with the transcription start site as well as its interactions with downstream sequences. 

### 2.6. Analysis of Intron Removal Events Enhanced by Anti-N1 

**Figure 6:**
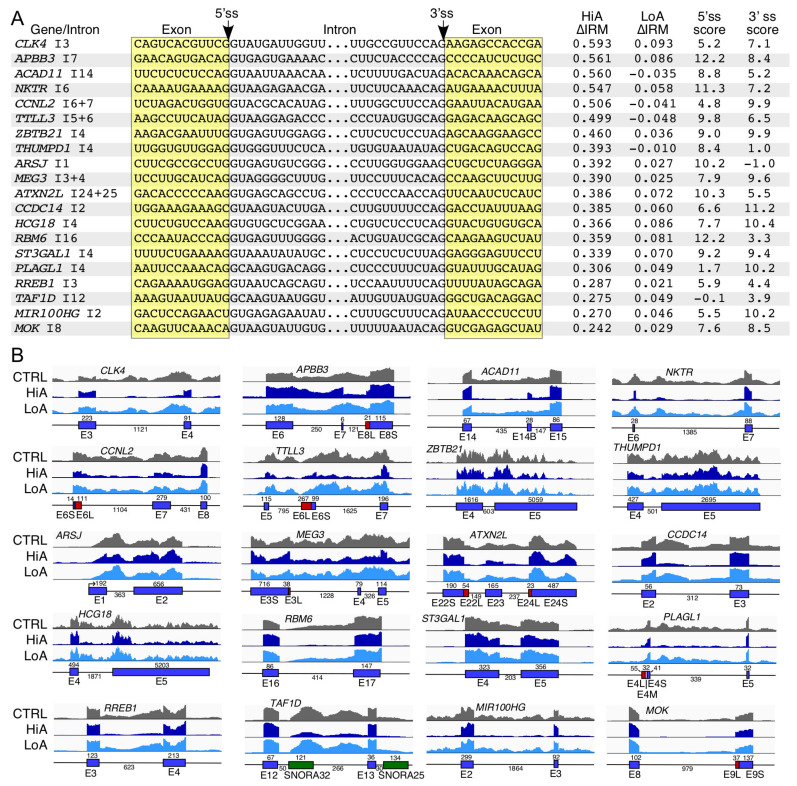
Increased intron removal caused by Anti-N1 treatment. (**A**). The sequence context of 20 candidate introns exhibiting greatly increased removal upon Anti-N1 treatment; 12 nucleotide-long sequences upstream and downstream of each indicated splice site of the affected introns are shown. ∆IRM indicates the change in proportional intron removal vs. intron retention. Other table contents and annotation are the same as in Figure 3A. (**B**). Genomic views of several candidate introns with increased removal after Anti-N1 treatment. In exons with 3 alternative 5′ or 3′ss, the intermediate region is colored pink. Other labeling and coloring are the same as in Figure 3C.

Enhanced intron removal (IRM) results from the efficient pairing between the 5′ss and 3′ss of an intron. HiA treatment triggered 618 IRM events (Supplementary Table S9) while LoA triggered 9 (Supplementary Table S10). We examined 20 IRM events caused by HiA treatment (Figure 6A). Genes associated with IRM events described here are linked to the following pathological conditions: cancer (*APBB3*, *RREB1*, *RBM6*, and *MIR100HG*), Fanconi anemia (*CLK4*, *CCNL2*, and *TTLL3*), cardiovascular defects (*ACAD11*, *ZBTB21*, *ST3GAL1*, and *ARSJ*), Noonan syndrome 1 (*THUMPD1*), neurological diseases (*ATXN2L*, *MOK*, *MEG3*, and *CCDC14*), metabolic disorders (*ATXN2L*, *MEG3*, and *PLAGL1*), and theileriasis (*NKTR*). Important cellular functions associated with genes affected by IRM include regulation of transcription (*APBB3*, *ZBTB21*, *RREB1*, *TAF1D*, *PLAGL1*), pre-mRNA splicing (*RBM6*), rRNA processing (*THUMPD1*), immune response (*NKTR*), cell signaling (*CLK4*, *MOK*, *CCNL2*), and metabolic pathways (*ACAD11*, *ST3GAL1*, *TTLL3* and *ARSJ*). Introns undergoing IRM triggered by HiA treatment had varied contexts. For example, both short and long introns were affected. Generally, the scores of the 5′ss and 3′ss of introns undergoing IRM events were lower than the average scores of the constitutively spliced introns (Figure 6A). In most cases, IRM triggered by Anti-N1 did not affect the removal of adjacent introns. However, there were few instances (see *CLK4* intron 3, *NKTR* intron 6, *CCNL2* intron 6, *TTLL3* intron 5, *ATXN2L* intron 24, and *CCDC14* intron 2) when an IRM event was associated with the improved removal of adjacent introns as well (Figure 6B). As expected, such events resulted in increased inclusion of an exon located between two introns associated with IRM. Similar to the IRT events, the IRM events are expected to be defined by an intron definition model. 

### 2.7. Analysis of Alternative Splice Usage Enhanced by Anti-N1

**Figure 7:**
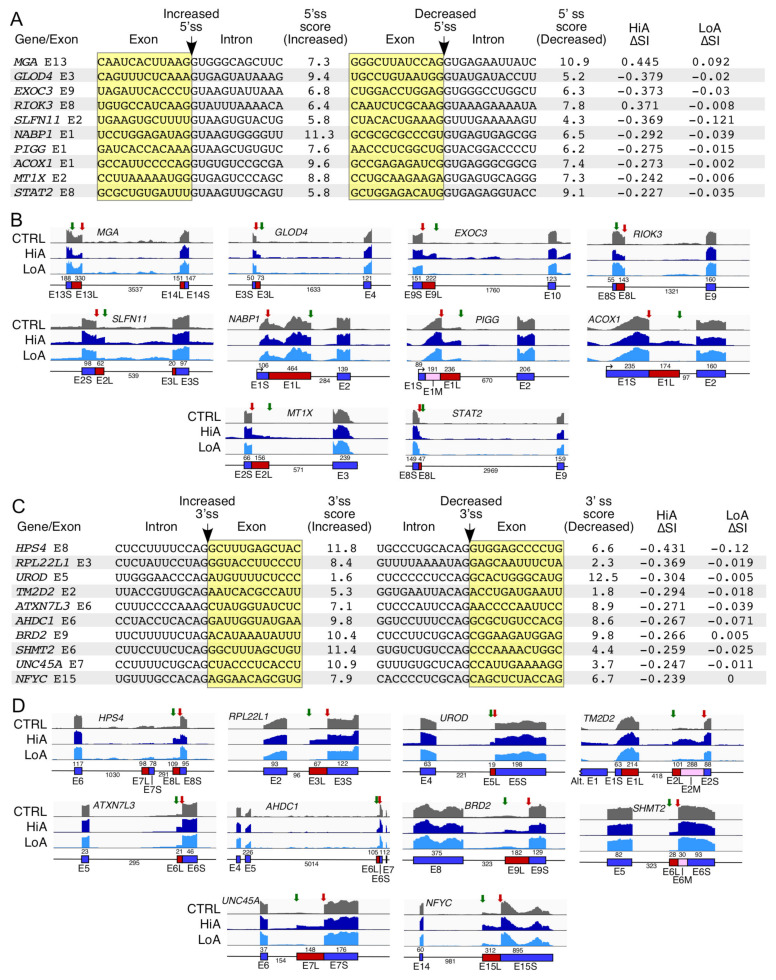
Alternative 5′ss and 3′ss usage caused by Anti-N1 treatment. (**A**). The sequence context of two alternative 5′ss of 10 candidate exons whose usage either increased or decreased upon Anti-N1 treatment. At the right of each sequence, the predicted strength of each 5′ss is shown. ∆SI indicates the change in proportional usage of the shorter isoform upon Anti-N1 treatment. Other coloring and labeling are the same as in Figure 3A. (**B**). Genomic views of the alternative 5′ss shown in panel A. Red arrows indicate the 5′ss with decreased usage, green arrows, the 5′ss with increased usage. Other labeling and coloring are the same as in Figure 3C and Figure 6B. (**C**). The sequence context of two alternative 3′ss of 10 candidate exons whose usage either increased or decreased upon Anti-N1 treatment. Other table contents and annotation are the same as in Figure 3A. (**D**). Genomic views of the alternative 3′ss shown in panel C. Red arrows indicate the 3′ss with decreased usage, and green arrows the 3′ss with increased usage. Other coloring and labeling are the same as in Figure 3C and Figure 6B.

Alternative usage of splice sites changes the sizes of exons and has potential to alter the coding reading frames of a protein. Between alternative 5′ss (A5S) and 3′ss (A3S), HiA caused a combined 842 alternative events and LoA caused 5 (Supplementary Tables S11–S14). We examined 20 representative events where the usage of the alternative 5′ss (A5S) and 3′ss (A3S) were affected by HiA treatments (Figure 7). Genes hosting A5S and A3S described here are associated with susceptibility to pathological conditions, such as cancer (*MGA*, *RIOK3*, *SLFN11*, *NABP1*, *RPL22L1*, and *NFYC*), mental retardation (*PIGG*), neurological disorders (*TM2D2*, *ATXN7L3*, *AHDC1*, and *BRD2*), metabolic disorder (*ACOX1*, *HPS4*, *UROD* and *SHMT2*), and infectious diseases (*MT1X*, *STAT2*, *SHMT2*, and *NFYC*) (Figure 7). These genes are also involved in critical cellular functions, including DNA binding (*AHDC1* and *BRD2*), transcription (*MGA*, *ATXN7L3*, and *NFYC*), receptor binding (*UNC45A*), exocytosis (*EXOC3*), cell signaling (*RIOK3*, *ACOX1*, *STAT2*, and *TM2D2*), tRNA binding (*SLFN11*), DNA replication and repair (*NABP1*), metabolic pathways (*PIGG*, *MT1X*, *UROD*, and *SHMT2*), lysosome biogenesis (*HPS4*), and ribosome assembly (*RPL22L1*) (Figure 7). 

We found no direct correlation between the scores of the splice sites and the incidences of the A5S or A3S events. Additionally, the usage of the alternative splice sites occurred in exons bordering both long and short introns. However, we observed a preference for inclusion of “longer” exons due to the usage of the distal splice sites (Figure 7B,D). In some instances, the size of the relatively short intron was further decreased by the A5S or A3S events. For example, in case of *ACOX1* and *RPL22L*, selection of A5S and A3S caused by HiA reduced the sizes of introns 1 and 2 from 271 and 163 nucleotides to 97 and 96 nucleotides, respectively (Figure 7B,D). In general, intron retention in the A5S and A3S events affected by HiA was mostly restricted to introns in which the alternative 5′ss or 3′ss resided. However, there were some notable exceptions. For example, in case of *EXOC3* intron 9 that harbored the alternative 5′ss, we also observed increased retention of the downstream intron 10 upon HiA treatment (Figure 7B). In case of *TM2D2*, the usage of the alternative 3′ss within intron 1 favored enhanced transcription from an upstream promoter, suggesting a co-transcriptional regulation of splicing (Figure 7D). Interestingly, the expression of *BRD2* that underwent an A3S event was also substantially upregulated. It is possible that the increase in transcription might favor the usage of the alternative 3′ss within *BRD2* intron 8.

### 2.8. Comparison of Perturbed Expression of Genes by ISS-N1-Targeting Splice-Switching Oligonucleotides

**Figure 8:**
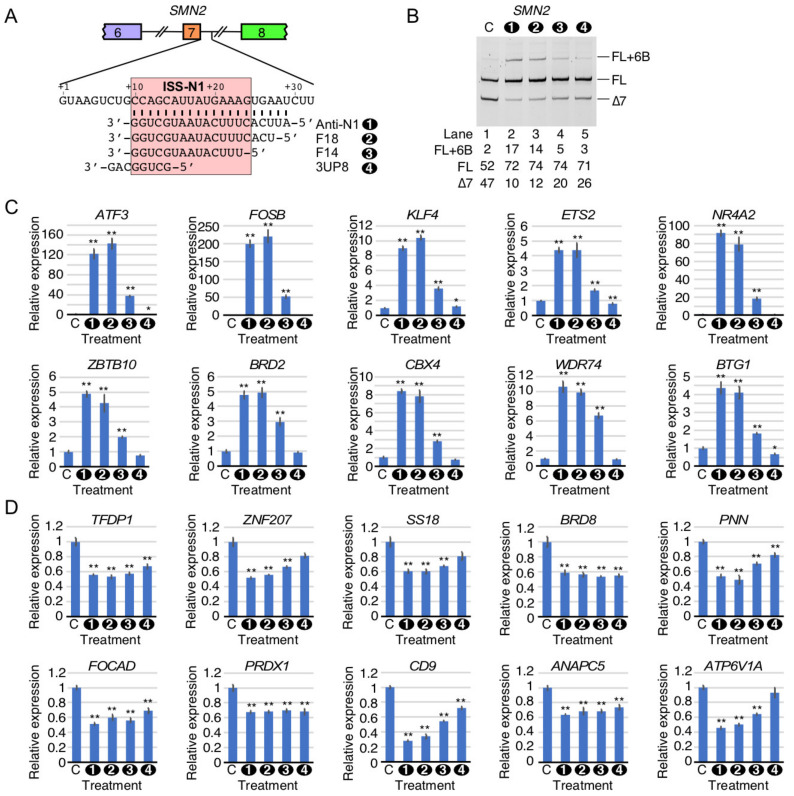
Comparison of perturbed expression of genes by ISS-N1-targeting splice-switching oligonucleotides. (**A**). Sequences and target binding sites of four Anti-N1 targeting splice switching oligonucleotides (ISOs). Labeling and coloring is the same as Figure 1A. For brevity and clarity, the ISOs are numbered 1–4 as indicated. (**B**). Effects of ISO treatment on *SMN2* splicing. Treatments are indicated at the top of the gel, with “C” signifying the treatment with the nontargeting control ASO. Splice isoform identity is indicated at the right side of the gel. The percent of total transcript for each splice isoform is indicated below the gel. (**C**). qPCR validation of the upregulated genes after ISO treatment. Gene symbols are shown at the top of each panel. Y axis: relative expression to non-targeting control. Treatments are indicated at the bottom of each graph, with “C” signifying the treatment with the nontargeting control ASO. Error bars represent the standard error of the mean. *n* = 3. *: *p* < 0.05. **: *p* < 0.01. (**D**). qPCR validation of the downregulated genes after ISO treatment. Labeling is the same as in panel C.

To validate the findings of RNA-Seq, we performed quantitative PCR (qPCR) on selected transcripts that showed perturbed expression by HiA. Our validation experiment included independent samples treated with the high concentrations of Anti-N1 as well as three additional ISS-N1-targeting splice-switching oligonucleotides (ISOs): F18, F14, and 3UP8 (Figure 8A). All four ISOs contain the same chemical modifications and are known to stimulate *SMN2* exon 7 inclusion [37,42]. While Anti-N1 anneals to the entire ISS-N1 and five downstream nucleotides, F18 anneals to the entire ISS-N1 plus three downstream nucleotides (Figure 8A) [42]. F14 anneals to the first 14 nucleotides of ISS-N1 (Figure 8A) [42]. 3UP8 is an 8mer ASO that targets the first five nucleotides of ISS-N1 as well as three nucleotides upstream of ISS-N1 (Figure 8A) [42]. 3UP8 is the shortest known ASO to stimulate *SMN2* exon 7 inclusion at low nanomolar concentrations [42]. Considering 3UP8 blocks only 1/3rd of the ISS-N1 sequence and requires sequestration of three upstream nucleotides for its high activity, its mechanism of action is expected to be distinct from the other three ISOs we employed here [42]. As expected, the treatment of GM03813 cells with 100 nM of all four ISOs promoted *SMN2* exon 7 inclusion, albeit with varying degrees (Figure 8B). In case of F18 and F14, we observed an off-target inclusion of cryptic exon 6B, although to a lesser extent than the 20mer Anti-N1 (Figure 8B). 3UP8 only slightly promoted exon 6B inclusion (Figure 8B).

We first validated the off-target effects of ISOs on the expression of ten transcripts that were found to be highly upregulated by HiA in our RNA-Seq analysis. These transcripts are *ATF3*, *FOSB*, *KLF4*, *ETS2*, *NR4A2*, *BRD2*, *CBX4*, *ZBTB10*, *WDR74*, and *BTG1* (Figure 8C). ATF3 is a transcription factor that responds to a broad variety of stressors [49]. In motor neurons, ATF3 is strongly induced after axonal injury [50]. After treatment with Anti-N1 or F18, *ATF3* levels were increased by >100-fold, while F14 induced ~40-fold upregulation (Figure 8C). In contrast, 3UP8 caused a slight downregulation of *ATF3* (Figure 8C). Fos proteins dimerize with Jun proteins to form transcriptionally active complexes [51]. Alternative splicing of *FOSB* can produce a splice isoform, ∆FosB, that is associated with neuronal plasticity and the development of addictions [52]. *FOSB* was upregulated ~200-fold in response to both Anti-N1 and F18, while F14 triggered a ~50-fold increase (Figure 8C). 3UP8, in contrast, did not affect *FOSB* levels at all (Figure 8C). KLF4 is a key transcriptional regulator that controls cell proliferation and differentiation through the p21protein encoded by the *CDKN1A* gene [53]. In addition, KLF4 controls pluripotency and is one of a few transcriptional regulators that can reprogram fibroblasts into induced pluripotent stem cells [54]. *KLF4* was upregulated ~9-fold by Anti-N1, ~10.4-fold by F18, and ~3.6-fold by F14 (Figure 8C). 3UP8 caused only a slight upregulation (~1.2-fold) of *KLF4* (Figure 8C). ETS2 is a transcription factor associated with cancer cell proliferation, due in part to its ability to induce *hTERT* transcription [55]. *ETS2* expression was increased by ~4.2-fold by both Anti-N1 and F18 and ~1.7-fold by F14 (Figure 8C). In contrast, expression was slightly reduced (~0.8-fold) by 3UP8 (Figure 8C). NR4A2 is a nuclear receptor protein associated with energy metabolism, inflammation, and neuronal differentiation and homeostasis [56]. *NR4A2* mutations are strongly linked to familial Parkinson’s disease [57]. *NR4A2* was strongly upregulated by Anti-N1 (~90-fold), F18 (~80-fold), and F14 (~18-fold) (Figure 8C). 3UP8 did not significantly affect *NR4A2* levels. ZBTB10 binds directly to telomere repeat variants and may play a tumor suppressive role through inhibition of Sp transcription factors [58,59]. *ZBTB10* levels were increased ~4.9-fold by Anti-N1, ~4.3-fold by F18, and ~2-fold by F14 (Figure 8C). 3UP8 had no effect on *ZBTB10* transcript levels. BRD2 is a bromodomain-containing protein that recognizes hyperacetylated histones in order to enhance transcription of target genes [60]. Of note, exon 9 of *BRD2* underwent alternative 3′ss selection after HiA treatment (Figure 7D). BRD2 was upregulated ~5-fold by Anti-N1 and F18 and ~3-fold by F14 (Figure 8C). 3UP8 did not affect *BRD2* transcript levels (Figure 8C). CBX4 is a polycomb group protein that associates with the core Polycomb repressive complex 1 (PRC1) in order to control heterochromatin formation [61]. *CBX4* was upregulated ~8-fold by Anti-N1 and F18 and ~2.8-fold by F14 (Figure 8C). *CBX4* levels were unaffected by 3UP8 (Figure 8C). WDR74 is a nucleolar ribosome maturation factor that participates in early cleavage of pre-rRNA [62]. *WDR74* was upregulated ~10-fold by Anti-N1 and F18 and ~6-fold by F14 treatment (Figure 8C). 3UP8 had no effect on *WDR74* expression. BTG1 is a tumor suppressor protein that plays key roles in cell proliferation and stress response by associating with transcriptional regulators and controlling mRNA stability of target genes [63]. *BTG1* was upregulated >4-fold by Anti-N1 and F18 ASOs and ~1.8-fold by F14. In contrast, *BTG1* was slightly downregulated (~0.7-fold) by 3UP8 (Figure 8C).

We next validated the expression of ten transcripts that were downregulated by HiA according to our RNA-Seq analysis. These transcripts included *TFDP1*, *ZNF207*, *SS18*, *BRD8*, *PNN*, *FOCAD*, *PRDX1*, *CD9*, *ANAPC5/APC5*, and *ATP6V1A*. TFDP1 interacts with E2F transcription factors to regulate cell proliferation and is upregulated in some cancers [64]. *TFDP1* was downregulated to a similar extent (~0.6-fold) by all four ISOs (Figure 8D). ZNF207 is a transcriptional regulator that is predicted to play a key role in self-renewal and pluripotency of embryonic stem cells [65]. *ZNF207* expression was reduced to 0.52 times its normal expression level by Anti-N1, while F18 reduced expression to 0.56 times and F14 to 0.67 times normal. Effect of 3UP8 on *ZNF207* expression was not statistically significant. SS18 is found in mammalian SWI/SNF complexes that participate in chromatin remodeling [66]. In synovial sarcoma, SS18 is commonly fused to a portion of the SSX protein, destroying its ability to act as a tumor suppressor [67]. *SS18* was downregulated by Anti-N1 or F18 to 0.6 times and by F14 to 0.67 times its normal expression levels (Figure 8D). Its expression trended lower after 3UP8 treatment, but the difference was not statistically significant (Figure 8D). BRD8 is a bromodomain-containing chromatin remodeling protein that controls cell fate [68]. *BRD8* transcript expression was reduced to ~0.55–0.6 times normal levels by all four ISOs. PNN is a dual-function protein that plays roles in cell-cell adhesion and RNA regulation, being present at both desmosomes and in the nucleus as part of exon-junction complexes [69,70]. All four ISOs downregulated *PNN* levels with significantly stronger effects observed with the longer ISOs (Figure 8D). FOCAD is a focal adhesion protein that has tumor suppressor properties and genetic variants of FOCAD are associated with Alzheimer’s disease [71]. All four ISOs caused small but statistically significant downregulation of *FOCAD* expression. PRDX1 is a key signal transducer in response to extracellular hydrogen peroxide, catalyzing the formation of disulfide bonds in downstream proteins in the presence of H_2_O_2_ [72]. *PRDX1* transcript levels were reduced to ~0.65 times by all four ISOs (Figure 8D). CD9 is a tetraspanin-type cell surface molecule that is found in specific types of extracellular vesicles [73]. In addition, CD9 in the oocyte plasma membrane plays an indispensable role in fertilization [74]. *CD9* showed a clear size-dependent decrease by ISOs, ranging from a ~4 fold decrease by Anti-N1 to ~0.7 times normal expression by 3UP8 (Figure 8D). ANAPC5/APC5 is a member of the anaphase promoting complex, a large ubiquitin ligase that controls cell cycle progression [75]. *ANAPC5* was downregulated to 0.63–0.74 times normal expression by all four ISOs (Figure 8D). ATP6V1A is a subunit of the vacuolar H+-ATPase (v-ATPase) that controls lysosomal pH, enabling its ability to degrade its contents [76]. *ATP6V1A* transcript levels were reduced to ~0.4–0.6 times normal expression by Anti-N1, F18, and F14 treatment in a size-dependent manner, and were unaffected by 3UP8 (Figure 8D).

### 2.9. Comparison of Perturbed Splicing Events by ISS-N1-Targeting Splice-Switching Oligonucleotides

**Figure 9:**
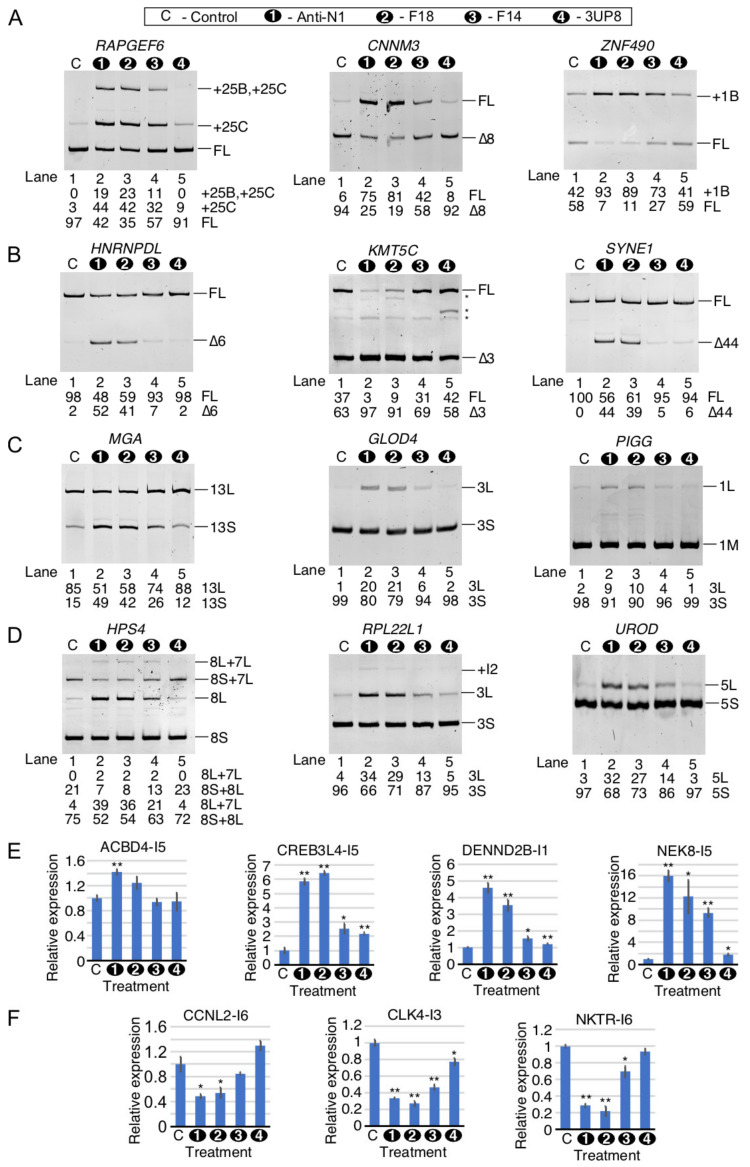
Comparison of perturbed splicing events by ISS-N1-targeting splice-switching oligonucleotides. (**A**). Validation of enhanced exon inclusion (EIN) events triggered by ISO treatments. Representative ethidium bromide-stained gels depicting semiquantitative RT-PCR of *RAPGEF6*, *CNNM3*, and *ZNF490* capturing the splicing event of interest. The gene name and the type of ISO treatment are indicated at the top of each gel. Similar to Figure 8A, the ISOs are numbered 1–4 and “C” signifies the treatment with the nontargeting control ASO. Splice isoform identity is indicated at the right side of the gel. Asterisks (*) indicate minor non-specific PCR products. The percent of total transcript for each splice isoform is indicated below each gel. (**B**). Validation of enhanced exon skipping (ESK) events triggered by ISO treatments. Representative ethidium bromide-stained gels depicting semiquantitative RT-PCR of *HNRNPDL*, *KMT5C*, and *SYNE1* (to capture the relevant splicing event). Labeling is the same as in panel A. (**C**). Validation of alternative 3′ss (A3S) usage upon ISO treatments. Representative ethidium bromide-stained gels depicting semiquantitative RT-PCR of *MGA*, *GLOD4*, and *PIGG* (to capture the relevant splicing event). Labeling is the same as in panel A. (**D**). Validation of alternative 5′ss (A5S) usage upon ISO treatment. Representative ethidium bromide-stained gels depicting semiquantitative RT-PCR of *HPS4*, *RPL22L1*, and *UROD* (to capture the splicing event of interest). Labeling is the same as in panel A. (**E**). qPCR validation of enhanced intron retention (IRT) events in *ACBD4*, *CREB3L4*, *DENND2B*, and *NEK8* in transcripts. Labeling is the same as in Figure 8C. (**F**). qPCR validation of enhanced intron removal (IRM) events in *CCNL2*, *CLK4*, and *NKTR* in transcripts. N = 3. *: *p* < 0.05. **: *p* < 0.01. Labeling is the same as in Figure 8C.

We validated the off-target splicing events that were captured in our RNA-Seq analysis of HiA-treated SMA patient cells. For validations of exon inclusion/skipping and alternative splice site usage, we used semi-quantitative PCR. The PCR-amplified products were resolved on a native polyacrylamide gel. Here, again, we compared the effects of four ISOs described above. RAPGEF6 is a guanine nucleotide exchange factor for RAP1 small GTPase that is critical for neurogenesis in the cerebral cortex [77]. HiA induced inclusion of two unannotated exons in between exons 25 and 26 of *RAPGEF6*, which we called exons 25B and 25C (Figure 3C). As compared with the control, we observed an increase in inclusion of exon 25C with or without inclusion of 25B to more than 50% in the presence of Anti-N1 or F18, while F14 treatment resulted in a more moderate inclusion (43%) (Figure 9A). 3UP8 treatment produced only a slight increase in exon 25C inclusion but no apparent inclusion of exon 25B. CNNM3 is a magnesium import protein that has oncogenic properties [78]. *CNNM3* exon 8 is predominantly skipped in GM03813 fibroblasts, whereas HiA promoted exon 8 inclusion (Figure 3C). Consistently, Anti-N1, F18 and F14 promoted *CNNM3* exon 8 inclusion in our validation assay, with effect being more pronounced with the longer ISOs (Anti-N1 and F18). Changes in splicing of *CNNM3* exon 8 were insignificant upon 3UP8 treatment (Figure 9A). Similar results were observed with exon 1B of *ZNF490* that codes for a zinc finger transcription factor of unknown function (Figure 9A). The multifunctional hnRNPDL protein is associated with regulation of transcription and alternative splicing [79]. Mutations in the prion-like domain of hnRNPDL cause muscular dystrophy-like conditions [80]. HiA was found to trigger skipping of exon 6, resulting in an in-frame deletion of the C-terminal prion-like domain of the hnRNPDL protein (Figure 3C). Consistently, we observed increase in skipping of *hnRNPDL* exon 6 caused by Anti-N1 and F18, whereas F14 and 3UP8 produced small and no changes in *hnRNPDL* exon 6 splicing, respectively (Figure 9B). KMT5C is a lysine methyltransferase that methylates lysine 20 on histone H4 in order to mediate chromatin compaction and regulate DNA repair [81]. In agreement with the results of RNA-Seq (Figure 4), *KMT5C* exon 3 skipping was increased by Anti-N1, F18, and F14 with the effect being more pronounced with the longer ISOs (Figure 9B). The effect of 3UP8 on *KMT5C* exon 3 splicing was similar to that of the control ASO (Figure 9B). SYNE1 is a nuclear envelope protein that participates in the control of nucleus positioning [82]. Critically, loss of SYNE1 activity results in aberrant organization of synaptic nuclei and myonuclei, causing severe defects of motor neuron innervation [82]. Supporting the findings of RNA-Seq (Figure 4C), Anti-N1 and F18 induced substantial skipping of *SYNE1* exon 44 (Figure 9B). We observed only negligible effects of F14 and 3UP8 on splicing of *SYNE1* exon 44 (Figure 9B).

Next, we examined the alternative splice site (A5S and A3S) usage triggered by the ISOs. MGA is a transcription factor that dimerizes with MAX, another transcription factor of the same family, in order to activate transcription of a wide variety of target genes. Exon 13 of the *MGA* mRNA has two potential 5′ss [83]. In agreement with the findings of RNA-Seq (Figure 7B), Anti-N1 promoted usage of an upstream 5′ss of *MGA* exon 13 (Figure 9C). The effect was also observed with F18 and F14 in a size-dependent manner, whereas 3UP8 produced no appreciable changes in splicing of *MGA* exon 13 (Figure 9C). GLOD4 is a poorly characterized homolog of GLO1, which is required for detoxification of methylglyoxal, a toxic byproduct of glycolysis [84]. Supporting the findings of RNA-Seq (Figure 7B), the usage of a downstream 5′ss of *GLOD4* exon 3 was induced by Anti-N1 (Figure 9C). Similar effects were observed with F18 and F14 in a size-dependent manner, whereas 3UP8 had no appreciable effect on splicing of *GLOD4* exon 3 (Figure 9C). PIGG functions in the production of glycosylphosphatidylinositol (GPI) modified proteins in order to allow them to anchor to the plasma membrane [85]. Loss of function mutations in the *PIGG* gene are linked to developmental delay and epilepsy [85]. The results of RNA-Seq revealed induction of a downstream 5′ss by HiA treatment, extending the size of the first exon (1L) (Figure 7B). Inclusion of 1L is predicted to produce a shorter protein, due to usage of a downstream AUG as a start codon during translation. Consistent with the findings of RNA-Seq, Anti-N1 promoted inclusion of *PIGG* exon 1L (Figure 9C). Similar effects were observed with F18 and F14 in a size-dependent manner, whereas 3UP8 produced no appreciable changes in splicing of *PIGG* exon 1 (Figure 9C).

HPS4 participates in the biogenesis of melanosomes, platelet dense granules, and lysosomes, and mutations in HPS4 result in Hermansky-Pudlak syndrome characterized by albinism, excessive bleeding, and pulmonary fibrosis [86]. *HPS4* displays a complex splicing pattern, including alternative 3′ss usage and skipping of exon 7 and alternative 3′ss usage of exon 8 (Figure 7D). The results of our RNA-Seq revealed an increased usage of an upstream 3′ss of exon 8, producing a longer exon 8 that we call 8L (Figure 7D). Inclusion of *HPS4* exon 8L is predicted to result in disruption of the full-length protein due to introduction of a frame-shift. In our validation experiment, inclusion of *HPS4* exon 8L was induced to ~35–40% by Anti-N1 and F18 and ~20% by F14. At the same time, we observed no significant change in splicing of *HPS4* exon 8 upon 3UP8 treatment (Figure 9D). RPL22L1 is a paralog of the ribosomal protein RPL22 and can function similarly in translation [87]. However, in some cases, such as in hematopoietic stem cell development, these proteins can have distinct and even antagonistic functions [88]. Based on the results of RNA-Seq, HiA triggers the enhanced usage of an alternative 3′ss upstream of *RPL22* exon 3, forming a longer exon 3 (3L) (Figure 7D). Confirming the findings of RNA-Seq, Anti-N1 and F18 increased the level of *RPL22* exon 3L-containing transcripts to 34% and 29%, respectively, along with a slight increase in intron 2 retention (Figure 9D). While F14 also promoted the usage of the alternative 3′ss of *RPL22* exon 3, albeit to a lesser extent, we observed no appreciable effect of 3UP8 on *RPL22* exon 3 splicing (Figure 9D). UROD participates in the biosynthesis of heme in mammals, and mutations in UROD cause familial porphyria cutanea tarda, which results in skin fragility and blisters [89]. Consistent with the findings of RNA-Seq (Figure 7D), Anti-N1 and F18 promoted the usage of an alternative 3′ss of *UROD* exon 5, forming a longer exon 5 (5L) (Figure 9D). F14 produced a somewhat decreased effect on *UROD* exon 5L 3′ss usage, whereas 3UP8 had no appreciable effect on *UROD* exon 5 splicing (Figure 9D). 

We used qPCR to confirm the off-target effects of ISOs on intron retention events triggered by HiA (Figure 9E,F). ACBD4 is a peroxisomal protein that mediates association of peroxisomes with the endoplasmic reticulum [90]. Supporting the findings of RNA-Seq, we observed a 1.4-fold increase in retention of *ACBD4* intron 5 upon Anti-N1 treatment (Figure 9E). Other three ISOs had no significant effect on *ACBD4* intron 5 retention (Figure 9E). CREB3L4 is a member of the CREB/ATF family of transcription factors that is highly expressed in male germ cells and plays a role in spermatogenesis [91]. In agreement with the findings of RNA-Seq, Anti-N1 and F18 triggered a ~6-fold increase in *CREB3L4* intron 5 retention (Figure 9E). However, F14 and 3UP8 produced a much more settled effect on *CREB3L4* intron 5 retention (Figure 9E). DENND2B is found in the periphery of cells and can control cell morphology and motility, potentially leading to metastasis [92]. The *DENND2B* gene is highly heterogenous, having more than 10 potential transcription start sites. HiA treatment triggered retention of the first intron of the predominant transcript that we observed in GM03813 fibroblasts (Figure 5C). Validating the findings of RNA-Seq, the results of qPCR showed intron 1 retention in samples treated with the ISOs, with the effect being much more pronounced in case of Anti-N1 and F18 (Figure 9E). NEK8 is a member of the NEK family of protein kinases that are widely involved in microtubule organization and cell division [93]. In particular, NEK8 is essential for cilia function [93]. Validating the findings of RNA-Seq, the results of qPCR showed a ~16-fold increase in *NEK8* intron 5 retention in the presence of Anti-N1 (Figure 9E). Other ISOs triggered *NEK8* intron 5 retention in a length-dependent manner (Figure 9E).

We also validated three improved intron removal events captured by RNA-Seq. CCNL2 is a member of the cyclin family of proteins that pairs with CDK11 in order to influence transcription and pre-mRNA splicing of downstream target genes [94]. Exons 6 through 8 of *CCNL2* undergo an array of alternative splicing events, including alternative 5′ss selection of exon 6, skipping/inclusion of exon 7, and intron retention of both introns 6 and 7. Supporting the findings of RNA-Seq (Figure 6B), we observed a ~2-fold decrease in *CCNL2* intron 6 retention by Anti-N1 and F18 (Figure 9F). F14 and 3UP8 showed no significant effect on *CCNL2* intron 6 removal (Figure 9F). CLK4 is a CDC2-like kinase that regulates alternative splicing by phosphorylating downstream SR-type splicing factors [95]. Consistent with the findings of RNA-Seq, Anti-N1 and F18 showed ~3-fold reduction in *CLK4* intron 3 retention (Figure 9F). The effect of F14 and 3UP8 *CLK4* intron 3 retention was small but noticeable (Figure 9F). The NK-TR protein is an immune response receptor that confers natural killer-like activity to T cells [96]. Validating the findings of RNA-Seq, results of qPCR showed a ~3-fold reduction in *NKTR* intron 6 retention caused by Anti-N1 and F18 (Figure 9F). The effect of F14 was less pronounced, while 3UP8 treatment had no impact on *NKTR* intron 6 removal (Figure 9F).

## 3. Discussion

ISS-N1, a unique regulatory sequence present in the human genome, is the target of the first FDA approved ASO-based therapy for the treatment of SMA [5,6]. The discovery of ISS-N1 was enabled in part by the employment of Anti-N1, a 20-mer ASO that substantially restored *SMN2* exon 7 inclusion by masking ISS-N1 (Figure 1A) [37]. Thus far, dozens of ISS-N1-targeting ASOs have been independently tested for their efficacies in vitro and/or in vivo [6,37,38,39,41,42,43,44,45,46,47,97,98,99,100,101,102,103,104,105,106]. Due to their tolerance for mismatches in base pairing, ASOs are expected to produce off-target effects, particularly when used at higher concentrations. However, predicting off-target effects caused by a splice-switching ASO employing currently available algorithms remains an impossible task. The nature and extent of off-target effects associated with any ISS-N1-targeting splice-switching oligonucleotide (ISO) has not yet been reported. Here, we present the analysis of transcriptome-wide changes that occur in SMA patient fibroblasts treated with a single dose of 5 nM (LoA) or 100 nM (HiA) concentration of Anti-N1. In order to create a head-to-head comparison between LoA and HiA, we adjusted LoA concentration to 100 nM with the non-targeting 10mer control. Our goal was to capture both sequence-dependent and sequence-independent off-target effects that occurred during the first 30 h of ASO treatments. Of note, ASO treatments for longer than 48h are associated with additional off-target effects [107]. LoA had a negligible off-target effect on the transcriptome of treated cells, yet, as expected, it produced a significant increase in *SMN2* exon 7 inclusion. HiA, on the other hand, substantially restored *SMN2* exon 7 inclusion, while at the same time causing massive perturbation of the transcriptome, affected the expression of thousands of genes. Expression of multiple types of genes, including coding, non-coding, and pseudogenes were impacted by HiA (Figure 1). In many instances, HiA caused more than a five-fold increase in the levels of abundantly expressed genes (Figure 1 and Figure 8). For downregulated genes, the HiA effect was less dramatic, as only a handful of the highly-expressed genes showed more than a 2-fold decrease (Figure 1 and Figure 8). Using qPCR, we validated ten upregulated and ten downregulated events captured by RNA-Seq (Figure 8). The genes affected by HiA are likely to impact all aspects of cellular metabolism, including DNA replication, DNA repair, transcription, pre-mRNA splicing, translation, macromolecular trafficking, cytoskeletal dynamics, signaling pathways, and cell cycle. 

Consistent with the massive perturbation of the transcriptome, HiA caused aberrations in seven types of pre-mRNA splicing events that we examined. These events included EIN, ESK, IRT, IRM, A3S, A5S, and MXE; dysregulations in each of them have potential to change a protein coding frame or/and produce a truncated protein (Figure 2). Using semi-quantitative PCR or qPCR, we validated 19 aberrant splicing events triggered by HiA (Figure 9). Partial annealing of Anti-N1 to splice sites and/or splicing regulatory cis-elements could be one of the major drivers of aberrant (off-target) splicing triggered by this ASO (Supplementary Figure S1). Depending on the location of its “partial” annealing, Anti-N1 could displace and/or recruit splicing factor(s). It is also possible that Anti-N1 could trigger structural rearrangements in pre-mRNA. The role of an RNA structure in pre-mRNA splicing regulation is a growing area of interest [40,108,109,110,111], and an ASO-based approach has been employed to abrogate structural elements critical for splicing control [39,112]. Off-target sites of Anti-N1 are expected to be randomly distributed throughout pre-mRNAs with their high prevalence in introns due to their (introns’) large size. Given the greater incidence of EIN events, we infer that more intronic negative elements than intronic positive elements are being targeted by Anti-N1. The comparatively low incidence of aberrant ESK events could be attributed to the likely low number of positive cis-elements that possess some degree of complementarity to Anti-N1. Of note, G-rich motifs have been implicated in splice site selection and intron removal [113]. Considering the 5′ end of Anti-N1 has extensive complementarity with G-rich motifs through both G:C (canonical) and G:U (wobble) base pairing, removal of introns harboring G-rich motifs are likely to be affected by Anti-N1. 

A combinatorial control, exerted by cis-elements spread across a given exon and their flanking intronic sequences, regulates exon inclusion/skipping [114,115,116,117,118]. As per the exon definition model, both splice sites of an internal exon must be defined before the removal of any of its flanking introns [114]. Furthermore, in the case of the exon definition model that is generally applicable to small exons flanked by large intronic sequences, cross-exon interactions are critical for defining an exon. On the contrary, cross-intron interactions provide the basis for the intron definition model that is mostly applicable when introns are small [115]. Importantly, there is not a clear cut-off point with respect to exon or intron sizes that could be implemented for either the exon or intron definition model. In principle, these two simplistic models are not mutually exclusive, as events can alter the nature of the mechanism used for exon inclusion and intron removal. While exons undergoing EIN and ESK events are likely to be governed by the exon definition model, IRT and IRM events are regulated by intron definition model. The findings of our study provide some insights into the mechanism of splicing regulation of a vast number of genes, particularly those with the retained introns. Of note, transcripts with retained introns generally remained in the nucleus and may serve some regulatory role [119]. There, the abnormally high levels of intron-retained transcripts may affect the availability of splicing factors by sequestering them and preventing their use in the removal of other introns. Therefore, it is likely that the enhanced EIN/ESK/IRM events alter the “availability” of splicing factors that otherwise would be utilized for intron removal. 

In addition to aberrant splicing, other mechanisms appear to be involved in perturbation of the transcriptome. For example, substantially higher expression of certain transcripts could be due to upregulation of transcription. Splicing factors are recruited co-transcriptionally, and the rate of transcription determines the outcome of splicing [120]. In certain instances, we observed changes in genes’ transcription start sites with a likely impact on splicing. Other dynamics, including direct interaction with DNA, binding to DNA-interacting proteins, and/or lncRNAs could account for the modulation of transcription by Anti-N1. Transcript levels are also modulated by NMD that degrades mRNAs harboring premature stop codons [121]. Hence, downregulation of some of the transcripts, particularly those undergoing ESK and IRT, could result from NMD due to acquisition of the premature stop codons. Anti-N1 has complementarity to a G-rich motif (GUGGGGG) present at the 3′-end of U1 snRNA, a key component of U1 snRNP that defines the 5′ss of an exon [122]. U1 snRNP also interacts with transcription machinery to regulate splicing and polyadenylation [123,124]. Therefore, a potential Anti-N1 interference in U1 snRNP biogenesis could have multiple adverse effects on transcription, splicing, and polyadenylation. Anti-N1 could affect transcription indirectly by binding to microRNAs and tRNAs that control the translation of transcription factors. Anti-N1 may also perturb translation of specific transcription factors by diverting their corresponding mRNAs to stress granules. Other indirect effects of Anti-N1 could be due to its potential interactions with factors involved in the metabolic pathways responsible for the biosynthesis of building blocks of proteins and nucleic acids. 

Increasing the size of an ISO is known to enhance the efficacy of *SMN2* exon 7 splicing correction, particularly at low nanomolar concentrations [42,44]. However, it is not known how changes in the size of ISOs would impact the off-target effects. We performed a comparative study to determine if a length of an ISO contributes towards the off-target effects (Figure 8 and Figure 9). While most of the off-target effects observed with the 20mer Anti-N1 remained the same with the 18mer ISO (F18), there was a substantial reduction in off-target effects when the 14mer ISO (F14) was used. Of note, the size and sequence of F18 were identical to that of nusinersen (Spinraza™) currently used for SMA treatment [6]. Oligonucleotides encompassing phosphorothioate backbone are known to produce off-target effects due to their interaction with cellular proteins [125,126,127]. However, since off-target effects were substantially minimized with shorter ISOs, we attribute sequence and the large size of ISOs in addition to the phosphorothioate backbone as the drivers of off-target effects. Future experiments employing ASOs of varying sizes and matched controls would address how size and sequence impacts the nature of off-target effects. Based on our results, we propose that the portion of ISOs that anneals to the 3′ half of ISS-N1 together with downstream sequences contributes the most towards the off-target effects. Consistently, the 8mer ISO (3UP8) that anneals to the 5′ portion of ISS-N1 and three upstream nucleotides had no significant off-target effects in the majority of examined cases (Figure 8 and Figure 9). However, in the absence of RNA-Seq data for shorter ISOs, our results should be interpreted with caution. Chemical modifications of ASOs may also alter the nature of their off-target effects [128]. Keeping in mind that we performed this study using ASOs with 2′OMe modifications, our results cannot be directly extrapolated to SMA drug nusinersen that contains MOE modifications. Further, tissue-specific effects of nusinersen are likely to vary given its differential body-wide distribution upon intrathecal mode of delivery currently employed for SMA treatment. However, limitations of an ASO-based therapy cannot be discounted unless concerns of transcriptome-wide perturbations are appropriately investigated and mitigated. On an optimistic note, our findings suggest that low dose of an ISS-N1-targeting ASO produces limited off-target effects on gene expression, including pre-mRNA splicing. Several small molecules capable of correcting *SMN2* exon 7 splicing have shown promise for SMA therapy and one has already been approved for the treatment of SMA [35,129,130,131,132]. These advancements are consistent with the growing need to develop additional therapies for SMA [133]. Future studies will be required to determine if the long-term efficacy of the low dose of an ISS-N1-targeting ASO could be improved by combining it with small molecule-based therapies aimed at correction of *SMN2* exon 7 splicing. 

## 4. Materials and Methods

### 4.1. Cell Culture and ASOs

All tissue culture media and supplies were purchased from Life Technologies (Waltham, MA, USA). GM03813 primary SMA patient fibroblasts were obtained from Coriell Cell Repositories (Camden, NJ, USA) and were grown in minimal essential medium (MEM, Gibco, catalog # 10370) supplemented with 2 mM GlutaMAX-1 (Gibco, Waltham, MA, USA) and 15% fetal bovine serum (Gibco) at 37 °C under 5% CO_2_. All ASOs used in this study were obtained from Dharmacon (Lafayette, CO, USA). and encompassed phosphorothioate backbone and 2′OMe modifications throughout the entire sequence similarly as previously reported [37,38,39,42]. 

### 4.2. ASO Transfections

Transfections with ASOs were performed similarly as described earlier [98,99]. Briefly, GM03813 cells were plated at a density of 1.1 × 10^6^ cells per 10 cm cell culture dish 16 h prior to ASO treatments. Cells were transfected with 100 nM ASOs using Lipofectamine 2000 (Life Technologies) following the manufacturer’s instructions. In the case of LoA where the concentration of the experimental ASO was less than 100 nM, the total concentration was adjusted to 100 nM using nontargeting control ASO. Six hours after transfection, the culture media was replaced with fresh media. Twenty-four hours later, cells were collected for RNA isolation using TRIzol Reagent (Life Technologies). ASO sequences are as follows: Anti-N1: 5′-AUUCACUUUCAUAAUGCUGG-3′. Control: 5′-UUGCCUUUCU-3′. F18: 5′-UCACUUUCAUAAUGCUGG-3′. F14: 5′-UUUCAUAAUGCUGG-3′. 3UP8: 5′-GCUGGCAG-3′. 

### 4.3. Reverse Transcription and PCR (RT-PCR) and Quantitative PCR (qPCR)

cDNA was generated using SuperScript III reverse transcriptase (RT) (Life Technologies) following the manufacturer’s instructions. For cDNA synthesis an oligo(dT)_12–18_ primer (Life Technologies) and random primers (Promega, Madison, WI, USA) were employed to generate cDNA for semiquantitative PCR and qPCR, respectively. Generally, 0.5 µg of total RNA was used as template per 5 µL of RT reaction. PCR amplification in a 20 µL reaction was performed using Taq DNA polymerase (New England Biolabs, Ipswich, MA, USA) following the manufacturer’s instructions. All primers used in this study are listed in Supplementary Table S17. PCR products were separated on native polyacrylamide gels and visualized by ethidium bromide staining. Analysis and quantifications of splice products were performed using ImageJ software. qPCR was performed using PowerUp SYBR green master mix (Life Technologies) on a QuantStudio 3 (Thermo Fisher, Waltham, MA, USA) thermocycler according to the manufacturer’s instructions. Relative expression was determined using the ∆∆Ct method using *OAZ1* as the normalizing assay. All primers were obtained from Integrated DNA Technologies (Coralville, IA, USA).

### 4.4. Library Generation and RNA-Seq

TRIzol-isolated total RNA was characterized using an Agilent Bioanalyzer RNA nano chip to confirm RNA integrity (RIN ≥ 8); 1 µg of total RNA was then subjected to rRNA depletion using the NEBNext rRNA depletion kit v2 (Human/Mouse/Rat) and library generation using the NEBNext Ultra II Directional RNA library prep kit for Illumina. Libraries were barcoded for multiplexing using NEBNext Dual Index oligos for Illumina. Library size distribution was determined using an Agilent Bioanalyzer DNA 1000 chip and libraries were quantified using a Qubit fluorimeter. Libraries were pooled together and sequenced on an Illumina Novaseq 6000 with an S2 flow cell using a 100-cycle, paired-end protocol. RNA-Seq data used in this study are hosted on the NCBI Sequence Read Archive under the accession number SRP303455.

### 4.5. RNA-Seq Mapping and Bioinformatic Analysis

Reads from RNA-Seq were mapped to the human reference genome build GRCh38 using HISAT2 [134]. For differential expression, mapped reads were assigned to genes according to the Gencode v33 human transcriptome annotation [135], using the featureCounts script from the Subread software package [136], then expression was estimated using the DESeq2 R package [137]. For alternative splicing, mapped reads were analyzed by rMATS [138], using the Gencode v33 human transcriptome annotation. After identification of alternative splicing events, significant events were manually identified using the following criteria: they must exhibit a false discovery rate (FDR) <0.05, each alternative isoform must have at least 10 average junction reads supporting its usage in either sample group, and absolute value of the change in proportion spliced in (∆PSI) must be more than 0.1 for alternatively spliced exons or 0.05 for intron retention/removal.

Significantly enriched Kyoto Encyclopedia of Genes and Genomes (KEGG) [139] pathways were identified using WEBGestalt (webgestalt.org, accessed December 2020). Differentially expressed genes were categorized by gene type, chromosomal location, and number of transcripts per gene using Ensembl BioMart to filter gene lists. As a reference list, we used all the genes with measurable expression (at least 10 reads per sample) in GM03813 cells. Transcription factors with enriched targets in differentially expressed genes were identified using ChEA3 [140]. Splice site scores were calculated using the tools hosted at http://rulai.cshl.edu/new_alt_exon_db2/HTML/score.html, accessed in 10 December 2020. Enriched sequence motifs in alternatively spliced exons and flanking intronic sequences were identified using MEME [141].

## 5. Conclusions

Spinal muscular atrophy (SMA) is the first human disease for which an antisense oligonucleotide (ASO)-based therapy based on the successful prevention of skipping of an exon (*SMN2* exon 7) became available. The therapeutic oligonucleotide of SMA targets a 15-nucleotide long intronic negative element, ISS-N1, located immediately downstream of the 5′ss of *SMN2* exon 7. ISS-N1-targeting splice-switching oligonucleotides (ISOs) of varied lengths encompassing different chemical modifications have been employed in several reported studies. To the best of our knowledge, this is the first study to capture the off-target effects of an ISO. Here, we reported the wide-ranging off-target effects observed in SMA patient cells treated with 100 nM of Anti-N1, a 20mer oligonucleotide that anneals to the entire ISS-N1, as well as five nucleotides downstream of ISS-N1. Expression of genes associated with all aspects of cellular metabolism were impacted within the first 30 h of Anti-N1 treatment. Off-target effects observed with Anti-N1 were found to be reduced when the size of the ISOs was reduced. Our findings underscore the potential for further improvement of ASO-based therapy of SMA through appropriate selection of the ASO size that produces the minimum off-target effects within a broad concentration range used for the treatment. Our findings also suggest that low concentrations of an ISO produce negligible off-target effects and would likely become a powerful alternative when combined with other splicing modulating compounds. 

## Figures and Tables

**Table 1:** Acronyms used in this report.

Acronym	Full Name
2′OMe	2′-O-methyl ribose sugar modification
3′ss	3′ splice site
3UP8	8mer ASO that partially targets ISS-N1
5′ss	5′ splice site
A3S	Alternative 3′ splice site usage
A5S	Alternative 5′ splice site usage
Anti-N1	20mer ASO targeting ISS-N1
ASO	antisense oligonucleotide
EIN	Increased exon inclusion
ESK	Increased exon skipping
F14	14mer ASO that targets ISS-N1
F18	18mer ASO that targets ISS-N1
HiA	High concentration (100 nM) of Anti-N1
IRM	Improved intron removal
IRT	Increased intron retention
ISO	ISS-N1 targeting splice switching ASO
ISS-N1	Intronic splicing silencer N1
L2FC	log(2) fold change
LoA	Low concentration (5 nM) of Anti-N1
MOE	2′-O-methoxyethyl ribose sugar modification
MXE	Mutually exclusive exons and/or mixed events

## Data Availability

All data generated or analyzed during this study are included in this published article and its supplementary information files.

## Supplementary Materials

The following are available online at https://www.mdpi.com/article/10.3390/ijms22168378/s1, Figure S1: Potential base pairing between Anti-N1 and target skipped exons, Table S1: Upregulated genes after HiA treatment, Table S2: Downregulated genes after HiA treatment, Table S3: Exon inclusion (EIN) events triggered by HiA treatment, Table S4: Exon inclusion (EIN) events triggered by LoA treatment, Table S5: Exon skipping (ESK) events triggered by HiA treatment, Table S6: Exon skipping (ESK) events triggered by LoA treatment, Table S7: Intron retention (IRT) events triggered by HiA treatment, Table S8: Intron retention (IRT) triggered by LoA treatment, Table S9: Increased intron removal (IRM) events triggered by HiA treatment, Table S10: Increased intron removal (IRM) events triggered by LoA treatment, Table S11: Exons with altered 5′ splice sites (A5S) after HiA treatment, Table S12: Exons with altered 5′ splice sites (A5S) after LoA treatment, Table S13: Exons with altered 3′ splice sites (A3S) after HiA treatment, Table S14: Exons with altered 3′ splice sites (A3S) after LoA treatment, Table S15: Mutually exclusive/mixed exons (MXE) altered after HiA treatment, Table S16: Mutually exclusive/mixed exons (MXE) altered after LoA treatment, Table S17: List of primers used for validation of alternative splicing and differential expression
